# Fast and slow: Recording neuromodulator dynamics across both transient and chronic time scales

**DOI:** 10.1126/sciadv.adi0643

**Published:** 2024-02-21

**Authors:** Pingchuan Ma, Peter Chen, Elizabeth I. Tilden, Samarth Aggarwal, Anna Oldenborg, Yao Chen

**Affiliations:** ^1^ Department of Neuroscience, Washington University, St. Louis, MO 63110, USA.; ^2^ Ph.D. Program in Neuroscience, Washington University, St. Louis, MO 63110, USA.; ^3^ Master’s Program in Biomedical Engineering, Washington University, St. Louis, MO 63110, USA.

## Abstract

Neuromodulators transform animal behaviors. Recent research has demonstrated the importance of both sustained and transient change in neuromodulators, likely due to tonic and phasic neuromodulator release. However, no method could simultaneously record both types of dynamics. Fluorescence lifetime of optical reporters could offer a solution because it allows high temporal resolution and is impervious to sensor expression differences across chronic periods. Nevertheless, no fluorescence lifetime change across the entire classes of neuromodulator sensors was previously known. Unexpectedly, we find that several intensity-based neuromodulator sensors also exhibit fluorescence lifetime responses. Furthermore, we show that lifetime measures in vivo neuromodulator dynamics both with high temporal resolution and with consistency across animals and time. Thus, we report a method that can simultaneously measure neuromodulator change over transient and chronic time scales, promising to reveal the roles of multi–time scale neuromodulator dynamics in diseases, in response to therapies, and across development and aging.

## Teaser

Fluorescence lifetime enables comparison of neuromodulator dynamics across multiple time scales and individual animals.

## INTRODUCTION

Neuromodulators such as acetylcholine (ACh) and dopamine (DA) can reconfigure neural circuits and transform animal behaviors (*1*–*11*), and their misregulation is implicated in mental disorders (*12*–*19*). Recent research has demonstrated the importance of both transient and sustained change of neuromodulators, likely due to phasic and tonic neuromodulator release, for brain functions (*20*–*24*). For example, as animals learn to associate a cue with a subsequent reward, DA transient shifts from reward to cue, showing the importance of transient neuromodulator dynamics for behavior state transitions (*7*, *25*, *26*). Demonstrating the critical role of sustained change of neuromodulators, elevated baseline dopamine levels precede and predict hallucination-like behavior (*24*). Thus, to advance our understanding of the function of neuromodulators in animal behavior, we need methods to simultaneously capture both transient and sustained neuromodulator changes.

Although both transient and sustained neuromodulator changes are important, no method could simultaneously record both types of changes. Classical methods such as microdialysis and electrochemical methods allow comparison of neuromodulator concentration over long periods of time and between animals (*27*–*31*). However, these methods lack spatial resolution, temporal resolution, or chemical specificity. Fluorescence intensity–based optical reporters of neuromodulators are now transforming the field of neuromodulation due to their high spatial and temporal resolution (*32*–*36*). However, fluorescence intensity does not only respond to changing neuromodulator concentrations but also depends on excitation light power and sensor expression level, which varies across long time periods, between brain regions, and between animals. As a result, intensity measurement cannot be used to compare sustained change in neuromodulator concentrations across these domains. Therefore, an ideal method would combine the benefits of classical methods and fluorescence intensity–based sensors to enable measurement of both transient changes in neuromodulator concentration at high-resolution and sustained changes across time and animals.

Fluorescence lifetime imaging microscopy (FLIM) measurement of optical sensors could fulfil the requirement of such an ideal method. Fluorescence lifetime measures the time between excitation and light emission of a fluorophore and is therefore independent of sensor expression levels or fluctuation in excitation light power (*32*, *37*–*40*). FLIM has been used successfully to uncover spatiotemporal dynamics of intracellular signals and voltage with biosensors (*40*–*51*).

Most optical sensors of neuromodulators are derived from G protein–coupled receptors (GPCRs) for the specific neuromodulators, where the third intracellular loop is replaced by a single circularly permuted fluorescent protein (*34*–*36*). Whereas one can rationally design FLIM sensors based on Förster resonance energy transfer (FRET) (*40*, *45*–*48*, *52*–*57*), it is extremely hard to predict whether a single fluorophore-based sensor will show lifetime change (*58*). Most single fluorophore sensors change their absorption coefficient upon conformational change (*58*, *59*) and thus show no lifetime change. Although a few dyes and single fluorescent protein–based sensors show lifetime change (*41*–*44*, *49*–*51*), no GPCR-based single fluorophore sensors were reported to show lifetime responses. Thus, it is unclear whether any intensity-based neuromodulator sensors can display fluorescence lifetime change; nor is it known whether FLIM is a viable technique to reliably measure neuromodulator levels across excitation light powers, different individual animals, and chronic time periods.

Here, we report a method that can accurately measure both transient and sustained change in neuromodulators in living animals. We found fluorescence lifetime response in single fluorophore neuromodulator sensors based on GPCRs. To determine whether lifetime changes can be leveraged to study neuromodulation in vivo, we tested the probe with the largest dynamic range, the ACh sensor GRAB_ACh3.0_ (GPCR activation-based acetylcholine sensor 3.0) (*60*). We found that, similar to intensity, lifetime measurement of GRAB_ACh3.0_ is dose sensitive and can detect ACh dynamics with high spatial and temporal resolution. In contrast to intensity, lifetime measurement of endogenous ACh shows high consistency across individual animals, across imaging conditions, and across chronic time periods in vivo. Our results have broad implications beyond ACh sensors. Methodologically, these results demonstrate the power of FLIM for neuromodulator measurement and the value of making fluorescence lifetime-compatible neuromodulator sensors. Biologically, FLIM measurement of neuromodulator sensors enables us to simultaneously capture both acute and sustained changes of neuromodulators, promising to reveal the role of transient change and basal level of neuromodulator release in disease models, in response to therapies, and across development and aging.

## RESULTS

### Fluorescence lifetime responses of neuromodulator sensors

**Fig. 1.**
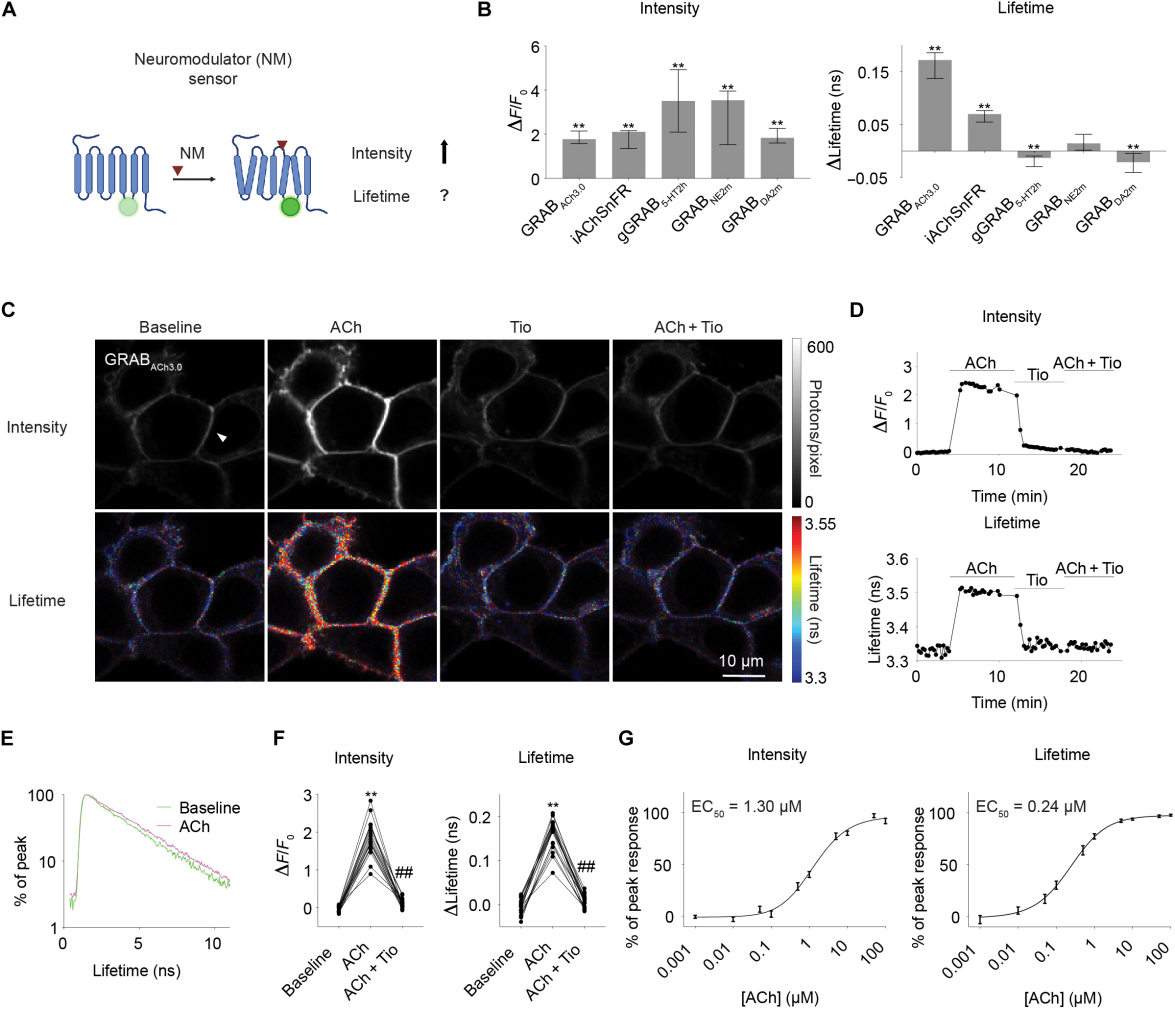
The ACh sensor GRAB_ACh3.0_ shows fluorescence lifetime response. (**A**) Schematic illustrating the question under investigation: Neuromodulator sensors show fluorescence intensity increase, but it is unclear whether they show any fluorescence lifetime change. The schematic was created with BioRender. (**B**) Summaries of fluorescence intensity and lifetime changes of different neuromodulator sensors in response to saturating concentrations of the corresponding neuromodulators in HEK 293T cells. Wilcoxon test, ^**^P < 0.01, versus baseline change. Data are represented as median with interquartile range. (**C** and **D**) Representative heatmaps (C) and traces (D) showing fluorescence intensity (top panels) or fluorescence lifetime (bottom panels) of GRAB_ACh3.0_ in response to saturating concentration of ACh (100 μM) with the cholinesterase inhibitor (AChEi) donepezil (Don; 5 μM), muscarinic ACh receptor (mAChR) antagonist tiotropium (Tio; 5 μM), or ACh + Tio + Don in HEK 293T cells. The traces in (D) are from the cell denoted by a triangle in (C). (**E**) Histogram of fluorescence lifetime of GRAB_ACh3.0_ sensor under baseline and with 100 μM ACh. (**F**) Summaries of intensity and fluorescence lifetime changes of GRAB_ACh3.0_ sensor in HEK 293T cells. Note that these data are the same as those displayed for GRAB_ACh3.0_ in (B). Friedman one-way analysis of variance (ANOVA) test with Dunn’s multiple comparison, **adjusted *P* < 0.01 versus baseline and ##adjusted *P* < 0.01 versus ACh. (**G**) Summaries of the dose-dependent intensity and fluorescence lifetime change of GRAB_ACh3.0_ sensor in response to different concentrations of ACh in the presence of 5 μM AChEi donepezil. Data are represented as mean with SEM. EC_50_, half maximal effective concentration.

We tested whether any intensity-based neuromodulator sensors showed a fluorescence lifetime change (Fig. 1A). We expressed individual sensors in human embryonic kidney (HEK) 293 T cells and measured sensor fluorescence intensity and lifetime with two-photon FLIM (2pFLIM). Unexpectedly, although not every sensor showed lifetime change, multiple sensors showed a significant fluorescence lifetime change in response to saturating concentrations of the corresponding neuromodulators [Fig. 1B; GRAB_ACh3.0_ (*60*), *n* = 18, *P* < 0.0001; intensity-based ACh-sensing fluorescent reporter (iAChSnFR) (*61*), *n* = 11, *P* = 0.001; 5-hydroxytryptamine (5-HT) sensor gGRAB_5-HT2h_ (*62*), *n* = 29, *P* = 0.0004; norepinephrine (NE) sensor GRAB_NE2m_ (*63*), *n* = 15, *P* = 0.1514; and DA sensor GRAB_DA2m_ (*64*), *n* = 19, *P* = 0.001). Notably, the ACh sensor GRAB_ACh3.0_, not previously optimized for lifetime, displayed a dynamic range of lifetime changes that are comparable to those of many FRET sensors (*46*–*48*, *52*–*57*). These results demonstrate that single fluorophore-based neuromodulator sensors can show fluorescence lifetime responses.

We subsequently used the ACh sensor GRAB_ACh3.0_ (*60*) to investigate the power of lifetime measurement because of the following reasons. First, GRAB_ACh3.0_ showed the largest fluorescence lifetime change among all the neuromodulator sensors tested (Fig. 1B; median of 0.17 ns with interquartile range of 0.14 to 0.19 ns in response to 100 μM ACh; *n* = 18, *P* < 0.0001). The large dynamic range makes it easier to explore the power of lifetime measurement in vivo. Second, ACh is one of the best-characterized neuromodulators. It increases during defined behavior state transitions, such as from resting to running (*60*, *65*–*67*) and from nonrapid eye movement (NREM) sleep to REM sleep (*60*, *68*–*73*), thus making it feasible to test the power of the technology with known ground truth. Third, ACh is one of the most important neuromodulators in the brain (*17*, *74*), playing critical roles in neuronal processes including learning and memory (*75*), attention (*76*), and sleep (*77*).

In the initial characterization of GRAB_ACh3.0_, similar to intensity, lifetime of GRAB_ACh3.0_ increased in response to saturating concentration of ACh (100 μM), and this increase was blocked by the addition of the muscarinic ACh receptor (mAChR) antagonist tiotropium (Tio; 5 μM) (*n* = 18, adjusted *P* = 0.0007 for intensity and *P* < 0.0001 for lifetime; ACh + Tio versus ACh; Fig. 1, C, D, and F). Furthermore, a mutant sensor that does not bind ACh (GRAB_ACh3.0mut_) did not show any intensity or fluorescence lifetime change in response to ACh (*n* = 5, *P* = 0.31 for intensity and 0.63 for lifetime; fig. S1). The fluorescence lifetime histogram of GRAB_ACh3.0_ showed slower decay with 100 μM ACh than without ACh at baseline (Fig. 1E), indicating that ACh binding increases fluorescence lifetime. Thus, both intensity and lifetime respond to ACh in cells expressing GRAB_ACh3.0_.

To test whether lifetime of GRAB_ACh3.0_ responds to graded ACh, we measured the dose-response curve of GRAB_ACh3.0_. In response to different concentrations of ACh ranging from physiologically relevant to saturating concentrations (1 nM to 100 μM) (*78*–*80*), fluorescence lifetime of GRAB_ACh3.0_ in HEK cells showed a dose-dependent increase (*n* = 13; Fig. 1G). In addition, fluorescence lifetime showed different sensitive concentration range to intensity [half maximal effective concentration (EC_50_) = 0.24 μM for lifetime and 1.30 μM for intensity; Fig. 1G]. These results indicate that lifetime measurement of GRAB_ACh3.0_ report graded ACh increase.

**Fig. 2.**
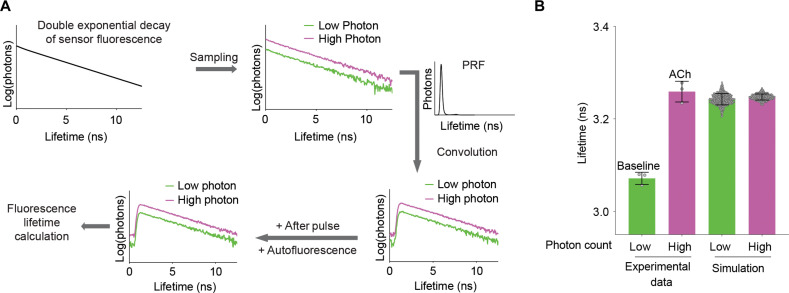
Simulation reveals authentic fluorescence lifetime response of GRAB_ACh3.0_. (**A**) Schematic illustrating the process of simulation. Fluorescence lifetime histogram of the sensor was modeled as a double exponential decay, sampled with different number of photons, and convolved with measured pulse response function (PRF). Subsequently, afterpulse and autofluorescence (sampled from measured distribution) were added. Empirical fluorescence lifetime was then calculated from the simulated distribution. (**B**) Fluorescence lifetime distribution of cells expressing GRAB_ACh3.0_ based on experimental data (*n* = 3) and based on simulation (*n* = 500 simulations under each condition). Experimental data were collected in the absence or presence of ACh (100 μM). Simulation assumed only intensity change, and no lifetime change of the fluorescence sensor, and simulated with low or high photon counts corresponding to baseline and ACh conditions, respectively. Data are represented as mean with SD.

In principle, an increase in fluorescence lifetime of cells expressing GRAB_ACh3.0_ could be due to true lifetime response to ACh by GRAB_ACh3.0_ or due to an increase in intensity of GRAB_ACh3.0_ relative to the autofluorescence of cells without any change of GRAB_ACh3.0_ lifetime. The latter possibility exists because both the fluorescent sensor and autofluorescence contribute to fluorescence measurement of cells, and the lifetime of GRAB_ACh3.0_ is longer than that of autofluorescence (fig. S2A). To test the null hypothesis that GRAB_ACh3.0_ showed no lifetime change, we performed computational simulations (*81*) to test how much cellular lifetime would increase if GRAB_ACh3.0_ only increased in intensity and not lifetime. For the simulation, we constructed photon populations of GRAB_ACh3.0_ sensor as double exponential decay (fig. S2B). Subsequently, we sampled from this population with low and high photon numbers corresponding to measurements at 0 and 100 μM ACh, respectively (Fig. 2A). We additionally added autofluorescence based on measurement in cells without sensor expression. Our simulation showed that if the sensor itself did not show any fluorescence lifetime increase, an increase in intensity only caused a small increase of overall lifetime (from 3.242 ± 0.012 ns to 3.247 ± 0.0065 ns; *n* = 500 simulations for both low and high photons; Fig. 2B). In contrast, the experimentally measured lifetime increased much more in response to 100 μM ACh (*n* = 3; mean difference = 0.19 ns; Fig. 2B): The increase was more than 10 times of the standard deviation (SD) (0.014 ns) of the difference between low and high photons from simulation. Therefore, the observed fluorescence lifetime response in cells expressing GRAB_ACh3.0_ is not solely due to an increase in fluorescence intensity. Rather, GRAB_ACh3.0_ sensor itself responds to ACh with authentic fluorescence lifetime increase.

### Fluorescence lifetime of ACh sensor detects graded and transient ACh change in the brain

**Fig. 3.**
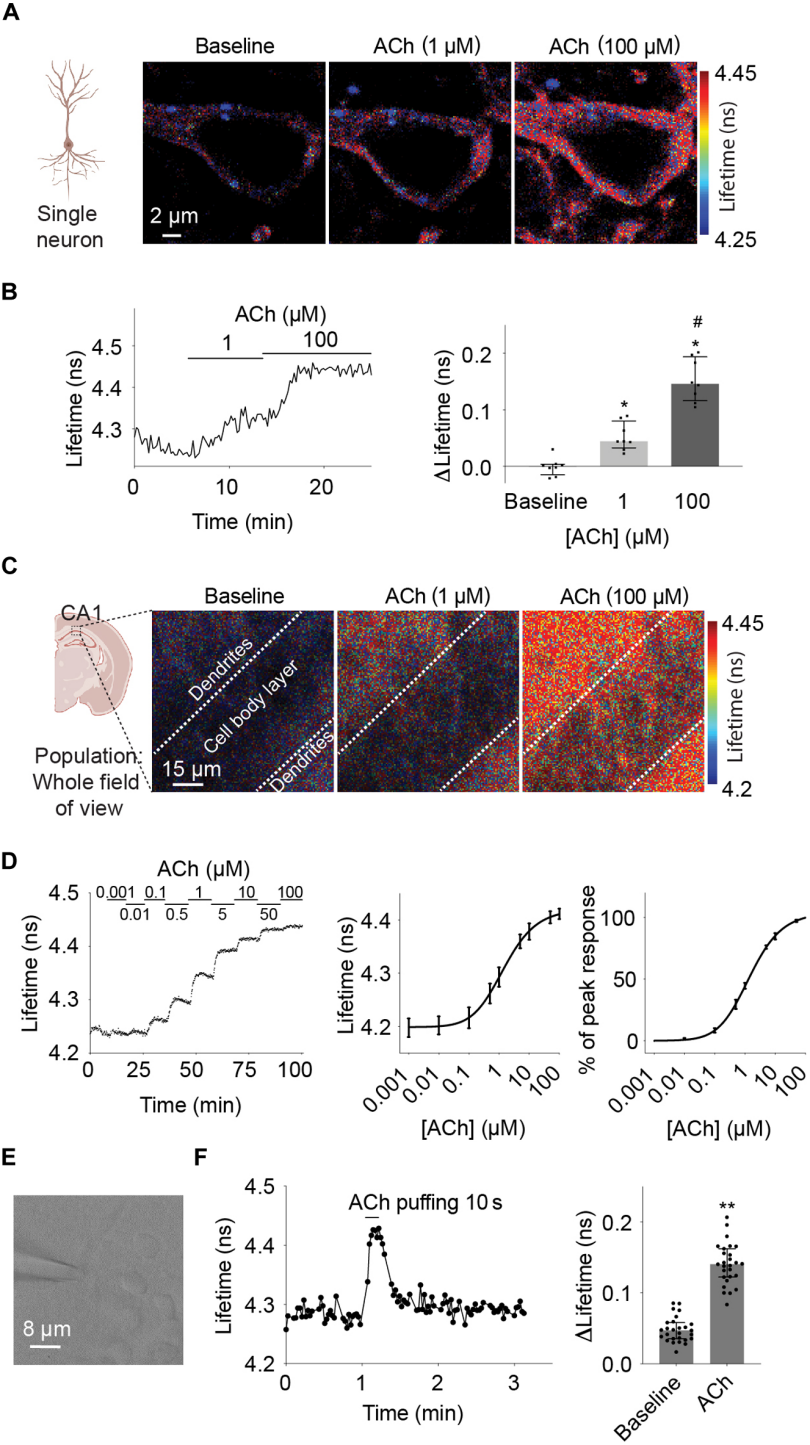
Fluorescence lifetime of GRAB_ACh3.0_ responds to graded and transient ACh in brain tissue. (**A** and **B**) Heatmaps (A), example trace, and summaries (B) showing fluorescence lifetime of individual hippocampal CA1 pyramidal neurons expressing GRAB_ACh3.0_ in response to ACh (1 and 100 μM, with 5 μM AChEi donepezil). Wilcoxon test with Bonferroni correction, *adjusted *P* < 0.05 versus baseline and #adjusted *P* < 0.05 versus 1 μM. Data are represented as median with interquartile range. (**C** and **D**) Heatmaps (C), example trace, and summaries (D) showing dose-response curve of fluorescence lifetime of a population of hippocampal CA1 neurons expressing GRAB_ACh3.0_ in response to various concentrations of ACh (with 5 μM AChEi donepezil). Data in (D) were from the whole field of view with a size of 90 μm by 90 μm. The summaries show the dose-response curve of the absolute fluorescence lifetime measurement (middle panel) and the percentage of the maximum response (right panel). Summary data in (D) are represented as mean with SEM. (**E**) Gradient contrast image showing puffing of ACh onto a CA1 pyramidal neuron with a glass pipette connected to a Picospritzer. (**F**) Example trace and summaries showing fluorescence lifetime of GRAB_ACh3.0_ in CA1 pyramidal neurons in response to a 10-s puff of ACh (200 μM). Wilcoxon test, **P < 0.01 versus baseline. Data are represented as median with interquartile range. Schematic illustrations from (A) and (C) were created with BioRender.

To test whether fluorescence lifetime of GRAB_ACh3.0_ can report ACh levels in brain tissue, we delivered the reporter via adeno-associated virus (AAV) injection to CA1 pyramidal neurons of the mouse hippocampus and imaged reporter responses in acute hippocampal slices. Bath application of ACh (1 μM and 100 μM) induced both fluorescence lifetime (*n* = 8 cells; adjusted *P* = 0.023 for baseline versus 1 μM, baseline versus 100 μM, and 1 μM versus 100 μM; Fig. 3, A and B) and intensity (*n* = 8; adjusted *P* = 0.023 for baseline versus 1 μM, baseline versus 100 μM, and 1 μM versus 100 μM; fig. S3, A and B) increase of GRAB_ACh3.0_. To mimic the response of GRAB_ACh3.0_ through an optical fiber in vivo, we also imaged whole fields of view of the CA1 region including populations of cell bodies and dendrites (Fig. 3C and fig. S3C). GRAB_ACh3.__0_ showed dose-dependent fluorescence lifetime (*n* = 5 fields of view; Fig. 3D) and intensity (fig. S3D, *n* = 5) responses to ACh. In addition, the absolute values of fluorescence lifetime correlated with ACh concentrations (Fig. 3D). These results indicate that fluorescence lifetime of GRAB_ACh3.0_ can report graded ACh increase in brain tissue.

For fluorescence lifetime measurement of GRAB_ACh3.0_ to be useful in biological applications, it needs to be sensitive enough to detect transient ACh in the brain. To test this, we puffed ACh (200 μM) onto the soma of CA1 pyramidal neurons in acute hippocampal slices (Fig. 3E) at temporal duration (10 s) comparable to ACh release measured in behaving animals in vivo (*82*). Both fluorescence lifetime (*n* = 27, *P* < 0.0001; Fig. 3F) and intensity (*n* = 27, *P* < 0.0001; fig. S3E) of GRAB_ACh3.0_ increased in response to ACh delivery, indicating that lifetime of GRAB_ACh3.0_ can report in brain tissue ACh release that is temporally relevant and transient. Together, these results show that similar to intensity, fluorescence lifetime of GRAB_ACh3.0_ can report graded and transient increase of ACh in the brain.

### Fluorescence lifetime of ACh sensor is independent of laser power

**Fig. 4.**
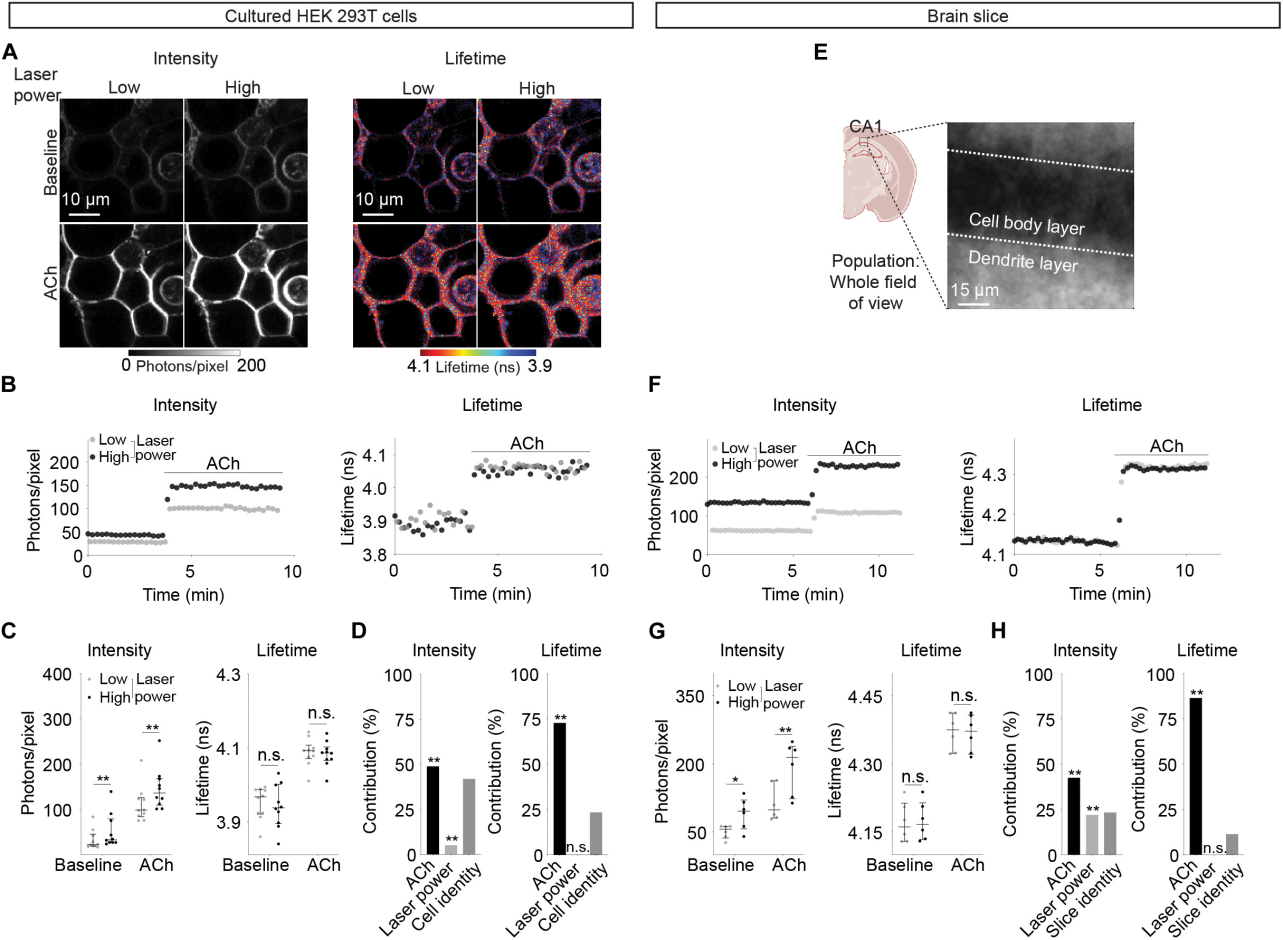
Fluorescence lifetime is stable across different excitation light powers. (**A** and **B**) Representative heatmaps (A) and traces (B) of intensity and fluorescence lifetime of HEK 293T cells expressing GRAB_ACh3.0_ in response to ACh (100 μM, with 5 μM AChEi donepezil), imaged at different laser powers. (**C**) Summaries of intensity and fluorescence lifetime of cells expressing GRAB_ACh3.0_ under different laser powers and in the absence and presence of ACh. Two-way ANOVA with Šídák’s multiple comparison, **adjusted *P* < 0.01, n.s., not significant; low versus high laser power. Data are represented as median with interquartile range. (**D**) Two-way ANOVA analysis showing the contribution to the total variance of the measurements due to ACh concentration, laser power, or cell identities. **P < 0.01. (**E**) Schematic and two photon image of a whole field of view (90 μm by 90 μm) of hippocampal CA1 pyramidal neurons expressing GRAB_ACh3.0_ in acute brain slices. The schematic was created with BioRender. (**F**) Representative traces of intensity and fluorescence lifetime of the whole field of view of hippocampal CA1 cells expressing GRAB_ACh3.0_ in response to ACh (100 μM, with 5 μM AChEi donepezil), imaged at different laser powers. (**G**) Summaries of whole fields of view intensity and fluorescence lifetime of hippocampal CA1 cells expressing GRAB_ACh3.0_ under different laser powers and in the absence and presence of ACh. Two-way ANOVA with Šídák’s multiple comparison, *adjusted *P* < 0.05 and **adjusted *P* < 0.01, low versus high laser power. Data are represented as median with interquartile range. (**H**) Two-way ANOVA analysis showing the contribution to the total variance of the measurements due to ACh concentration, laser power, or brain slice identities. **P < 0.01.

Unlike intensity, fluorescence lifetime should be independent of laser power fluctuation. To explore the extent of this advantage, we measured both fluorescence lifetime and intensity under different laser excitation powers, both in cultured HEK 293T cells and in brain slices. In 293T cells, we first evaluated whether the relative change of intensity or lifetime can reliably reflect change of ACh concentration despite varying laser powers. As laser power increased, the change of fluorescence lifetime in response to ACh remained consistent, whereas intensity change showed a small decrease under higher laser powers (*n* = 10; baseline: *P* = 0.055 for intensity and *P* = 0.71 for lifetime; ACh: *P* = 0.0003 for intensity and *P* = 0.95 for lifetime; fig. S4A). We subsequently evaluated whether absolute ACh concentration can be measured with sensor properties despite changing laser powers. As expected, fluorescence intensity of GRAB_ACh3.0_ increased with increasing laser power (*n* = 10; adjusted *P* = 0.0005 for baseline and *P* < 0.0001 for ACh, low versus high laser power; Fig. 4, A to C). Both laser power and the presence of ACh contributed significantly to the variability of fluorescence intensity across cells (*P* < 0.0001 for both ACh and laser power; Fig. 4D). Only 49% of sensor intensity variance could be explained by ACh concentrations (Fig. 4D). In contrast, fluorescence lifetime of the ACh sensor was stable across different laser powers (*n* = 10; adjusted *P* = 0.71 for baseline and 0.68 for ACh, low versus high laser power; Fig. 4, A to C). Only the presence or absence of ACh, and not laser power, significantly contributed to the variation of fluorescence lifetime across cells (*P* < 0.0001 for ACh and *P* = 0.12 for laser power; Fig. 4D). Notably, the majority (73%) of the variance of sensor lifetime could be explained by ACh concentration, with minimal contributions from laser power (0.11%) or cell identity (23%; Fig. 4D).

To test the stability of lifetime in brain tissue with varying laser excitation powers, we also imaged large fields of view in brain slices (Fig. 4, E to G). Whereas fluorescence intensity of GRAB_ACh3.0_ increased with increasing laser power (*n* = 6, adjusted *P* = 0.018 for baseline and *P* = 0.0052 for ACh, low versus high laser power; Fig. 4, F and G), fluorescence lifetime of the ACh sensor was stable across different laser powers (*n* = 6; adjusted *P* = 0.12 for baseline and *P* = 0.091 for ACh, low versus high laser power; Fig. 4, F to G). Whereas only 42% of sensor intensity variance could be explained by ACh concentration, the majority (87%) of the variance of sensor lifetime could be explained by ACh concentration (Fig. 4H). Together, these results indicate that fluorescence lifetime is a more reliable measurement of ACh concentration than fluorescence intensity under fluctuating laser powers.

### Fluorescence lifetime is consistent within a cell and between cells

If absolute fluorescence lifetime were to be used to predict ACh concentrations, then lifetime values would need to be stable within a cell for a given ACh concentration and consistent between cells. To test the stability of lifetime within a cell, we repeatedly applied ACh (1 μM). Similar to intensity, fluorescence lifetime was consistent within a cell across repeated application of the same concentration of ACh (*n* = 8; *P* > 0.99 for intensity and *P* = 0.95 for lifetime, first versus second flow-in; fig. S4, B and C). Thus, lifetime is consistent for a given ACh concentration within a cell.

**Fig. 5.**
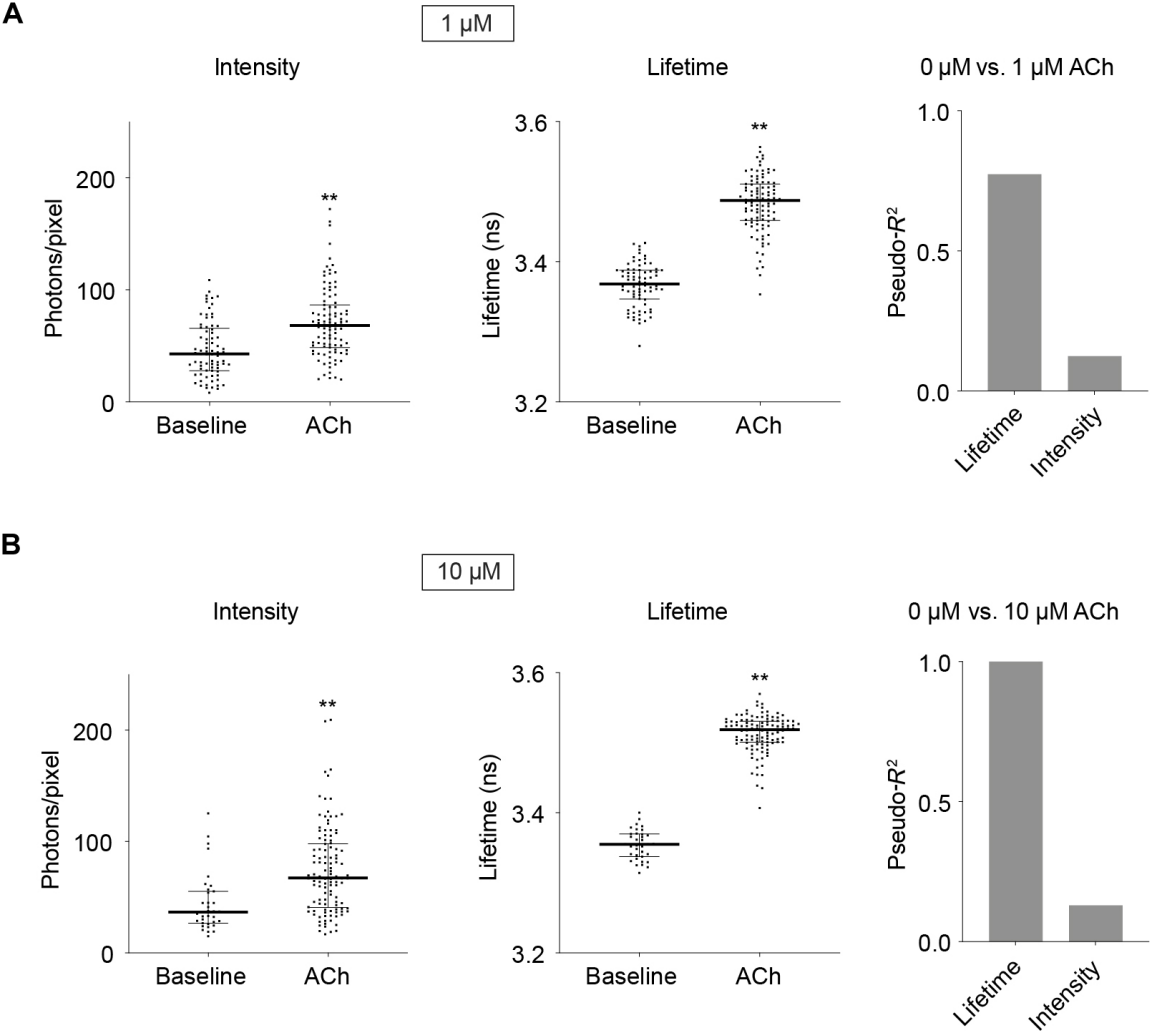
Fluorescence lifetime shows much less variability across cells and correlates better with ACh concentration than intensity. (**A** and **B**) Left: Distribution of intensity and fluorescence lifetime measurements of GRAB_ACh3.0_ in HEK 293T cells, at baseline, and with different concentrations of ACh (1 and 10 μM, with 5 μM AChEi donepezil). Mann-Whitney test, **P < 0.01 versus baseline. Data are represented as median with interquartile range. Right: Pseudo-*R*^2^ values between intensity/lifetime and ACh concentrations based on logistic regression, showing that lifetime measurement has much greater explanatory power than intensity for ACh concentration.

To test whether absolute fluorescence lifetime correlates well with ACh concentration between cells, we measured both lifetime and intensity exposed to a specified ACh concentration that is comparable to that reported in vivo (*78*–*80*). As expected, fluorescence intensity varied greatly between cells at a given ACh concentration [1 μM: coefficient of variation (CV) = 53.23% at baseline and 44.36% with ACh, *n* = 77 and 99; 10 μM: CV = 59.06% at baseline and 52.51% with ACh, *n* = 35 and 114; Fig. 5], likely due to different sensor expression levels across cells. Although fluorescence intensity increased in response to ACh (*P* < 0.0001 for baseline versus ACh, both 1 and 10 μM ACh; Fig. 5), intensity alone correlated poorly with ACh concentration [baseline versus ACh, pseudo-*R*^2^ (coefficient of determination) = 0.12 for 1 μM ACh and 0.13 for 10 μM ACh; Fig. 5]. In contrast, for fluorescence lifetime, variation between cells was much smaller (1 μM: CV = 0.91% at baseline and 1.17% with ACh, *n* = 77 and 99; 10 μM: CV = 0.63% at baseline and 0.75% with ACh, *n* = 35 and 114; Fig. 5). The signal-to-noise ratio for lifetime was thus higher. Absolute lifetime values correlated with ACh concentration with high accuracy (baseline versus ACh, pseudo-*R*^2^ = 0.77 for 1 μM ACh and pseudo-*R*^2^ = 1 for 10 μM ACh; Fig. 5). Similarly, in brain slices, the intensity values across CA1 neurons showed large variation (CV = 30.96% at baseline and 35.57% with 1 μM ACh, *n* = 23 and 30; fig. S5A), whereas the variation of fluorescence lifetime was much smaller (CV = 0.69% at baseline and 0.81% with 1 μM ACh; *n* = 23 and 30; fig. S5A). The variation of lifetime across cells was not due to the presence of varied amount of ACh at baseline (*n* = 13; *P* = 0.64 for baseline versus Tio; fig. S5B) or varied amount of cholinesterase activity [*P* = 0.67; CV = 1.12% without and 1.01% with cholinesterase inhibitor (AChEi) donepezil (5 μM); *n* = 40 and 61, respectively; fig. S5C]. The variability was comparable to the mutant sensor GRAB_ACh3.0mut_ that cannot bind ACh (*P* = 0.6041; CV = 0.79% without and 0.92% with ACh; *n* = 42 and 53 respectively; fig. S5D). These data suggest that lifetime variability between cells is likely due to the flexibility of sensor conformation. Furthermore, fluorescence lifetime, unlike fluorescence intensity, correlates with ACh concentration with high accuracy despite different sensor expression levels across individual cells.

### Fluorescence lifetime correlates with ACh-associated running-resting states with high accuracy across individual mice and varying excitation light powers

If a method can measure endogenous neuromodulator dynamics in vivo at multiple time scales, it needs to fulfill two criteria. (i) It should capture acute changes during rapid behavior state transitions. (ii) To capture sustained change, the measurement at the same neuromodulator concentration needs to be consistent across individual animals, imaging conditions, and chronic time scales. Although fluorescence lifetime should be robust, it can show variability due to conformational flexibility of the sensor or autofluorescence, and it has rarely been used to make comparisons across individual animals and weeks. To test whether lifetime measurement of GRAB_ACh3.0_ can fulfill these two criteria, we need to use known correlation between ACh and behavior states as ground truth. Here, we measured GRAB_ACh3.0_ across running-resting and sleep-wake states. ACh level is known to be higher during REM sleep, active wake (AW), and running and lower during NREM sleep, quiet wake (QW), and resting, respectively (*60*, *65*–*73*). These known ground truths allow us to perform proof-of-principle experiments to test whether lifetime can fulfill the criteria of an ideal method that can measure neuromodulator dynamics at multiple time scales.

We measured GRAB_ACh3.0_ in the hippocampus in freely moving mice via fluorescence lifetime photometry (FLiP) (*83*). FLiP measures the bulk fluorescence from a population of cells surrounding the tip of the fiber implant, allowing for the measurement of neuromodulator dynamics in genetically defined neurons in a brain region in vivo (*83*). The signal-to-noise ratio for the bulk signal is thus even higher than methods with cellular resolution. The variance of the lifetime from the bulk signal is inversely proportional to the number of cells. Thus, if the bulk signal of ~1000 cells were analyzed, the SD of lifetime distribution would be $\frac{1}{\surd 1000}∼\frac{1}{32}$  of the SD across single cells (fig. S6A), making FLiP a superb method to measure ACh level in vivo.

**Fig. 6.**
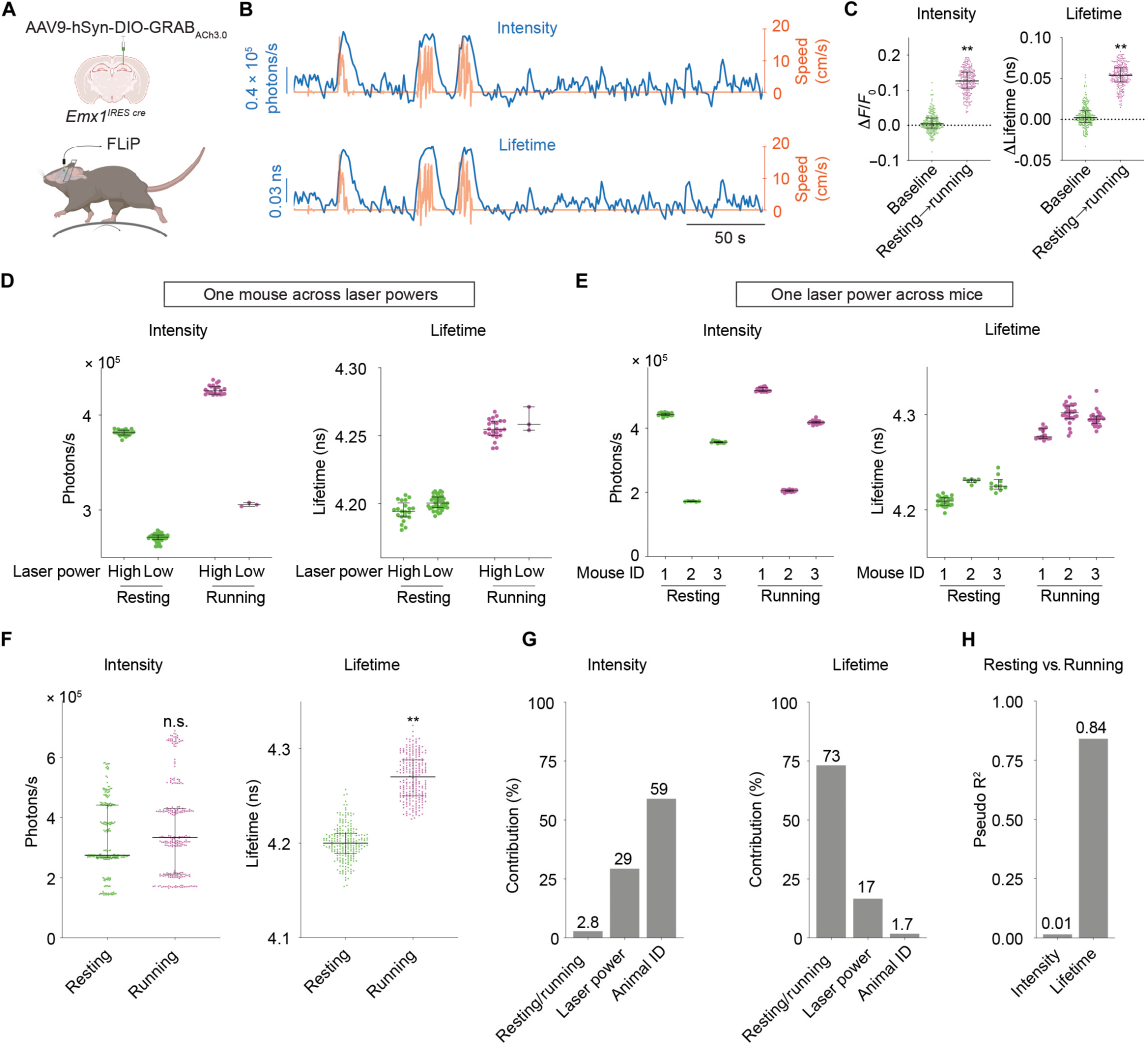
Fluorescence lifetime of GRAB_ACh3.0_ correlates with running versus resting states accurately despite varying laser powers and varying sensor expression levels across mice in vivo. (**A**) Schematic showing the experimental setup. AAV carrying Cre-dependent *GRAB_ACh3.0_* was delivered to CA1 cells in the hippocampus of *Emx1^IRES cre^* mice. FLiP was performed as head-fixed mice ran or rested on a treadmill. The schematic was created with BioRender. (**B**) Example traces showing intensity (top, blue) or fluorescence lifetime (bottom, blue) measurements from FLiP, and running speed (red) of GRAB_ACh3.0_-expressing mice on a treadmill. (**C**) Summaries of the change of intensity and lifetime of GRAB_ACh3.0_ within resting states and from resting to running. Data were pooled from different mice with different imaging laser powers. Nested *t* test, **P < 0.01. (**D**) Distribution of intensity and fluorescence lifetime of GRAB_ACh3.0_ in resting or running states from the same mouse but under different laser powers. (**E**) Distribution of intensity and fluorescence lifetime of GRAB_ACh3.0_ in resting or running states under the same laser power but from different mice. (**F**) Distribution of intensity and fluorescence lifetime of GRAB_ACh3.0_ in running or resting states, pooled from all mice across different laser powers (12 recordings from six mice under three different laser powers). Nested *t* test, **P < 0.01. (**G**) Results from stepwise-GLM analysis showing the contribution to the total variation of intensity or fluorescence lifetime of GRAB_ACh3.0_ from behavior states, laser power, and animal identities. Contribution was based on adjusted incremental *R*^2^. (**H**) Results from logistic regression analysis showing the power of explaining running or resting states with either intensity or fluorescence lifetime of GRAB_ACh3.0_, regardless of imaging laser powers or animal identities. Data are represented as median with interquartile range.

First, we tested whether fluorescence lifetime measurement of the ACh sensor can capture transient ACh increase as mice transitioned from resting to running. AAV virus carrying Cre-dependent *GRAB_ACh3.0_* was delivered to hippocampal CA1 region of *Emx1^IRES cre^* mice (*84*), labeling excitatory neurons and a subset of glia with the ACh sensor (Fig. 6A). We recorded fluorescence lifetime, intensity, and running speed simultaneously as mice voluntarily ran or rested on a treadmill (Fig. 6A). Both intensity and lifetime of GRAB_ACh3.0_ increased from resting to running (*n* = 233 running epochs, *P* < 0.0001 for intensity and *P* < 0.0001 for lifetime, baseline versus resting-to-running transition; Fig. 6, B and C). These results indicate that both properties capture transient ACh changes effectively. The increased intensity or lifetime from resting to running was not observed in control experiments with the mutant sensor GRAB_ACh3.0mut_ (fig. S6, B to D), indicating that the optical responses of GRAB_ACh3.0_ reflect endogenous release of ACh.

Second, we tested whether absolute values of lifetime can consistently report ACh concentrations across varying laser powers and across individual mice. These conditions mimic realistic scenarios because fluctuating laser power can arise from an unstable laser source or movement artifacts, and comparison across mice is essential if we want to compare wild type and disease models. Lifetime values during running did not correlate with running speed or duration of the running epochs (*n* = 233 running epochs; *P* = 0.29 for running speed and *P* = 0.13 for running duration; fig. S6, E and F). Thus, we treated all running epochs as the same state. Across varying laser powers, intensity showed large variation within the same behavioral state, whereas fluorescence lifetime remained remarkably stable (Fig. 6D). Similarly, with one laser power across different mice, intensity varied greatly within the same running or resting state, likely due to different sensor expression levels across mice. In contrast, lifetime remained stable within each state (Fig. 6E). When data from different imaging conditions and mice were combined, fluorescence intensity was not statistically different between running and resting (*n* = 226 resting epochs and 233 running epochs from 6 mice, *P* = 0.36; Fig. 6F), indicating that the absolute values of intensity could not be used to distinguish ACh levels between mice and between imaging conditions. Despite these differing conditions, lifetime showed significant increase from resting to running (*P* < 0.0001; Fig. 6F). These results indicate that in contrast to intensity, lifetime is consistent across imaging powers and across mice and can distinguish ACh-associated behavior states across these conditions.

To quantitate the power of fluorescence lifetime, we performed two statistical tests. First, we asked how much of the variance of lifetime and intensity could be explained by running versus resting states, laser power, and animal identity. For fluorescence intensity, most of the variance was explained by animal identity (59%), followed by laser power fluctuation (29%), with minimal variance explained by behavior state (2.8%) [adjusted incremental *R*^2^ of stepwise generalized linear model (stepwise-GLM); Fig. 6G]. In contrast, most of the variance in lifetime was explained by behavior state (73%), with small contributions from laser power (17%) and animal identity (1.7%) (adjusted incremental *R*^2^ of stepwise-GLM; Fig. 6G). Second, we performed logistic regression to ask how much we could explain running versus resting state solely based on lifetime or intensity. Lifetime showed much better explanatory power than intensity (pseudo-*R*^2^ = 0.84 for lifetime and pseudo-*R*^2^ = 0.01 for intensity; Fig. 6H). These results indicate that fluorescence lifetime, but not intensity, correlates with neuromodulator-associated behavior states despite fluctuating laser powers and expression level changes across animals. Together, although both intensity and lifetime of GRAB_ACh3.0_ capture acute neuromodulator changes effectively, lifetime excels when experiments call for comparison of neuromodulator levels across fluctuating laser powers and across animals.

### Fluorescence lifetime is consistent across chronic time scales

**Fig. 7.**
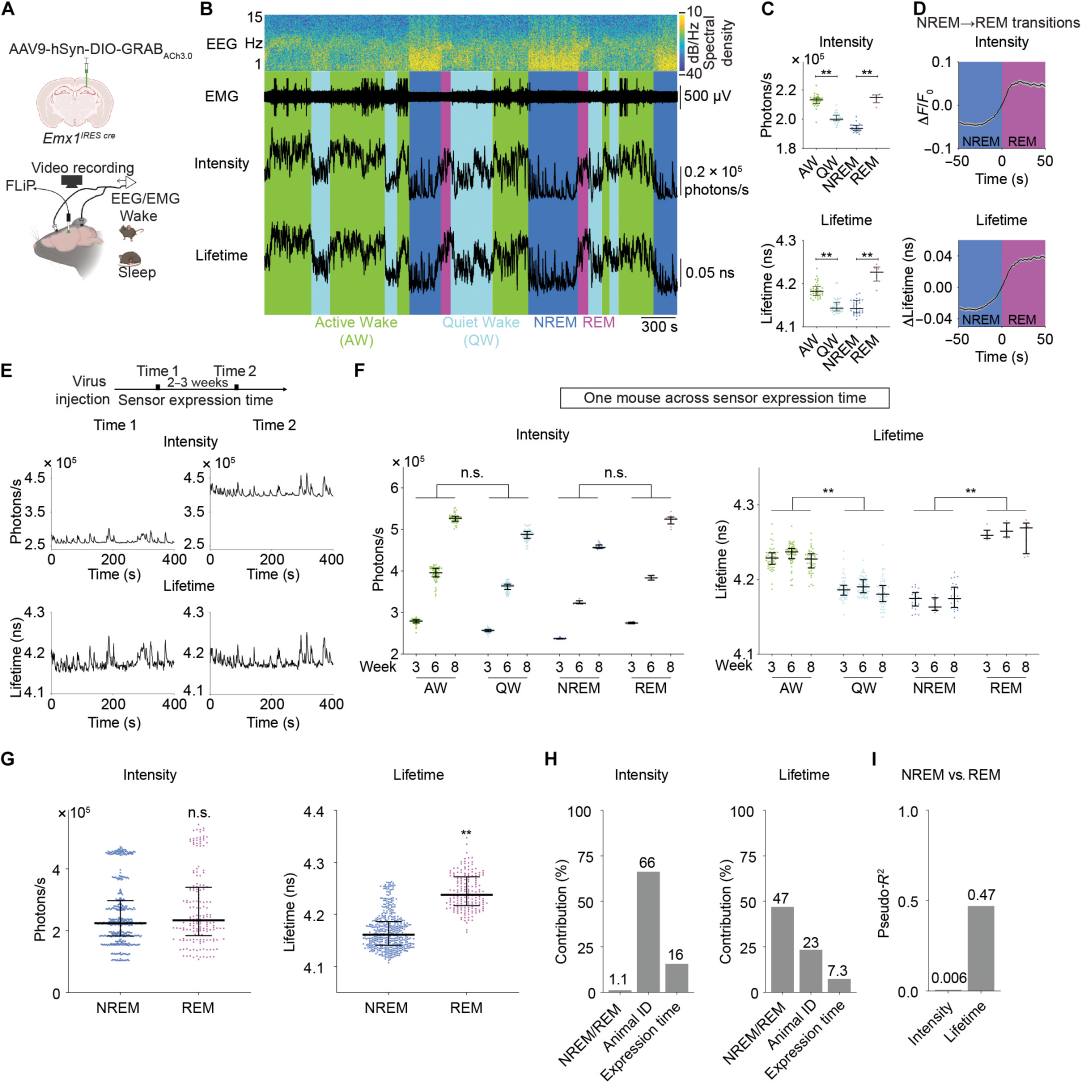
Fluorescence lifetime of GRAB_ACh3.0_ correlates with sleep-wake stages accurately despite variation in sensor expression levels across weeks and across animals. (**A**) Schematic showing the experimental setup. AAV carrying Cre-dependent *GRAB_ACh3.0_* was delivered to the hippocampal CA1 region of *Emx1^IRES cre^* mice. FLiP, EEG, EMG, and video recordings were performed across sleep-wake cycles over 9 hours in freely moving mice. The schematic was created with BioRender. (**B**) Example of EEG spectrogram, EMG trace, the scored sleep-wake states, as well as intensity and fluorescence lifetime traces from a mouse. (**C**) Distribution of intensity and fluorescence lifetime of GRAB_ACh3.0_ in different sleep-wake states from a 9-hour FLiP recording of one mouse. Kruskal-Wallis test with Dunn’s multiple comparison, **adjusted *P* < 0.01. (**D**) Summary traces of changes in intensity and fluorescence lifetime of GRAB_ACh3.0_ from NREM to REM sleep transitions. Data are represented as means with SEM. (**E**) Representative traces of intensity and fluorescence lifetime of GRAB_ACh3.0_ during NREM at two time points after virus injection. (**F**) Summaries of intensity and fluorescence lifetime of GRAB_ACh3.0_ in different sleep-wake stages in one mouse across sensor expression time. Nested t test, ^**^P < 0.01. (**G**) Distribution of intensity and fluorescence lifetime of GRAB_ACh3.0_ across NREM and REM sleep states, pooled from all mice across different sensor expression time (18 recordings from six mice at three sensor expression time points). Nested *t* test, **P < 0.01. (**H**) Results from stepwise-GLM analysis showing the contribution to the total variation of intensity or fluorescence lifetime of GRAB_ACh3.0_ from behavior states, sensor expression time, or animal identities. (**I**) Results from logistic regression showing the power of explaining NREM versus REM states with either intensity or fluorescence lifetime of GRAB_ACh3.0_, regardless of sensor expression time or animal identities. Other than (D), data are represented as median with interquartile range.

In vivo, the expression levels of a fluorescent sensor vary both across animals and across chronic time scales. We thus investigated whether fluorescence lifetime can accurately track ACh levels over many weeks, even as sensor expression levels change. We used sleep-wake cycles of mice as our proof-of-principle experiment. To evaluate the power of lifetime and intensity in explaining ACh-associated sleep and wake stages, we measured lifetime and intensity of the ACh sensor in the hippocampus with FLiP in freely behaving mice while simultaneously performing electroencephalogram (EEG), electromyography (EMG), and video recordings to determine sleep-wake stages (Fig. 7A).

We first asked whether lifetime, similar to intensity, reported acute changes of ACh as mice transitioned between different sleep-wake stages. For a given mouse recorded within a single day, both fluorescence lifetime and intensity of GRAB_ACh3.0_ increased from QW to AW and from NREM to REM sleep (*n* = 42, 42, 26, and 6 epochs for AW, QW, NREM, and REM respectively; adjusted *P* < 0.0001 for AW versus QW and NREM versus REM of both intensity and lifetime; Fig. 7, B and C). Both intensity and fluorescence lifetime change of ACh sensor could reliably detect ACh change associated with rapid sleep/wake stage transitions such as NREM to REM transitions (*n* = 217 transitions from six mice; Fig. 7D). These results indicate that fluorescence lifetime, similar to intensity (*60*), can detect acute ACh changes across sleep/wake stages.

To control for the specificity of the response, we performed the same experiment with the mutant ACh sensor GRAB_ACh3.0mut_ that does not bind to ACh (fig. S7, A to C). Unexpectedly, GRAB_ACh3.0mut_ showed an acute decrease in fluorescence intensity as mice transitioned from NREM to REM sleep (*n* = 42, 22, 50, and 14 epochs for AW, QW, NREM, and REM, respectively; adjusted *P* = 0.25 for AW versus QW and 0.0002 for NREM versus REM; fig. S7, A and B). Fluorescence lifetime did not show significant change between AW and QW or between NREM and REM (adjusted *P* = 0.46 for AW versus QW and 0.51 for NREM versus REM; fig. S7B), indicating that lifetime response of GRAB_ACh3.0 _during these behavior state transitions reflect changes in endogenous ACh release. Because the intensity of mutant ACh sensor responds to other environmental factors and not ACh, these data emphasize the importance of mutant sensor controls in the use of neuromodulator sensors.

To test the consistency of fluorescence lifetime as sensor expression level varies across long periods of time, after viral delivery of *GRAB_ACh3.0_*, we measured lifetime and intensity at three different time points that were weeks apart. We first determined whether acute ACh change upon behavior transitions can be stably detected over weeks. The changes of both GRAB_ACh3.0_ intensity and fluorescence lifetime from NREM to REM remained consistent (*n* = 61, 59, and 88 transitions for 3, 6, and 8 weeks after sensor expression, respectively; *P* = 0.15 for intensity and *P* = 0.25 for lifetime, across sensor expression time; fig. S7D), indicating that acute ACh change can be reliably detected by both intensity and lifetime. Second, we assessed how well the absolute values of fluorescence intensity and lifetime correlate with ACh levels that are associated with specific behavior states. As expected, fluorescence intensity showed marked changes over time (Fig. 7, E and F). When results were pooled across sensor expression time, intensity values were not significantly different between different behavior states (*n* = 169, 152, 48, and 18 total epochs for AW, QW, NREM, and REM, respectively; *P* = 0.77 for AW versus QW, and 0.61 for NREM vs. REM; Fig. 7F). In contrast, fluorescence lifetime remained remarkably stable for a given behavioral state, even as sensor expression changed over time (Fig. 7, E and F). Lifetime values were significantly different between behavior states despite sensor expression variation (*P* = 0.0007 for AW versus QW, and *P* < 0.0001 for NREM versus REM; Fig. 7F). Therefore, these results indicate that fluorescence lifetime, unlike intensity, is a consistent readout of ACh concentration over weeks and is strongly correlated with ACh-associated behavior states.

To ask whether lifetime correlates with ACh-associated NREM/REM states despite varying sensor expression levels across chronic time scales and across mice, we combined results from different sensor expression time and mice. Lifetime, unlike intensity, was still significantly different between NREM and REM sleep states (*n* = 444 NREM epochs and 183 REM epochs from 6 mice; *P* = 0.72 for intensity and *P* = 0.0006 for lifetime; Fig. 7G).

To quantitate the contributions to variation of lifetime and intensity by different factors, we calculated adjusted incremental *R*^2^ from stepwise-GLM. The variation of fluorescence intensity was largely explained by animal identity (66%), followed by sensor expression time (16%), with minimal contribution from behavior states (1.1%) (Fig. 7H). In contrast, lifetime variation was largely explained by NREM versus REM states (47%), with much less contribution from animal identity (23%) and sensor expression time (7.3%; Fig. 7H).

Conversely, we tested the extent to which lifetime or intensity could distinguish ACh-associated sleep stages. Lifetime showed much higher explanatory power for NREM versus REM states than intensity despite changing expression level and across different animals (pseudo-*R*^2^ = 0.006 for intensity and 0.47 for lifetime; Fig. 7I). Therefore, fluorescence lifetime is a better correlate of behavior state than intensity, when data from multiple animals and across weeks need to be considered.

Together, these results indicate that in vivo, fluorescence lifetime, similar to intensity, captures acute changes in neuromodulator levels within one animal. Fluorescence lifetime, and not intensity, correlates with neuromodulator levels and has much greater explanatory power than intensity when experiments call for comparison between animals and across long periods of time.

## DISCUSSION

**Fig. 8.**
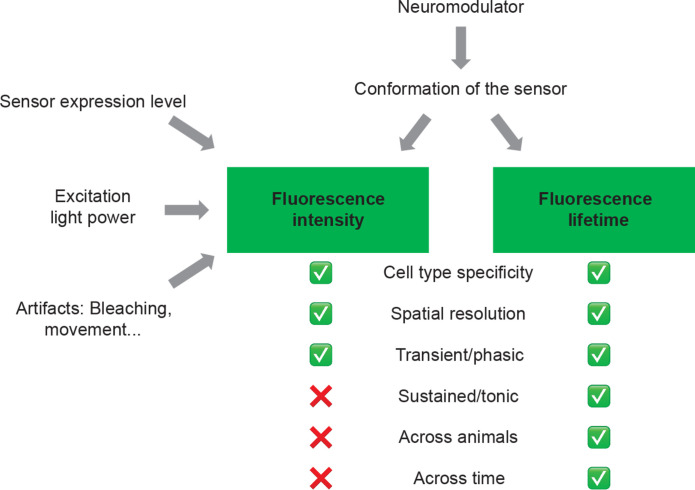
Comparison of intensity and lifetime measurement of fluorescent neuromodulator sensors. Fluorescence lifetime reflects conformation change of the sensor, whereas intensity is also influenced by sensor expression level, excitation light power, and other artifacts such as bleaching and movement. As a result, although fluorescence intensity enables measurements of neuromodulator concentrations with cell type specificity, high spatial resolution, and high temporal resolution to detect transient/phasic changes of neuromodulators, it cannot be used to compare sustained/tonic changes of neuromodulators and compare neuromodulator levels across animals or chronic time scale. Fluorescence lifetime, in contrast, excels in all these categories.

In summary, we found fluorescence lifetime responses for multiple neuromodulator sensors and thus reported a method that can accurately measure neuromodulator dynamics at multiple time scales. Similar to fluorescence intensity, fluorescence lifetime can detect transient neuromodulator changes and is dose sensitive. In contrast to fluorescence intensity, fluorescence lifetime is a consistent readout of neuromodulator concentration despite varying laser powers and with different sensor expression levels between cells. In vivo, we show that fluorescence lifetime, unlike intensity, consistently reports neuromodulator levels even as sensor expression level changes across weeks and across animals. Thus, fluorescence lifetime measurement of neuromodulator sensors opens doors to study neuromodulator dynamics both at high spatial and temporal resolution, and across animals, brain regions, and chronic time scale (Fig. 8).

### Advantages of using fluorescence lifetime to measure neuromodulator concentrations

When should we use lifetime over intensity measurement? On the basis of our results (Figs. 6 and 7), both lifetime and intensity can report acute (subsecond to second) and endogenous neuromodulator release in vivo. Fluorescence lifetime excels over intensity because lifetime measurement is independent of sensor expression (*32*, *37*–*40*). Because of this property, we demonstrate three major advantages of lifetime measurement in our proof-of-principle experiments. First, using behavior states as correlates of neuromodulator levels, we find that lifetime correlates with neuromodulator concentration with higher accuracy than intensity despite large variation of sensor expression levels over chronic time scale of weeks (Fig. 7), across individual animals (Figs. 6 and 7), and despite fluctuating excitation light power (Fig. 4 and Fig. 6). Second, absolute fluorescence lifetime correlates well with neuromodulator concentrations in brain slices (Fig. 3D), thus offering the potential of estimating absolute concentrations of ACh with lifetime measurement in vivo. Third, as demonstrated in our mutant sensor data, fluorescence lifetime is less prone than intensity to neuromodulator-independent change associated with NREM to REM transitions (fig. S7). This REM-associated intensity decrease calls for careful interpretation of data to distinguish neuromodulator change from other brain state-associated intensity change such as hemodynamic change.

What is the limitation of lifetime over intensity measurement? Accurate construction of fluorescence lifetime histogram requires a substantial number of photons (*81*). This necessitates longer integration time and lower sampling rates compared to intensity measurements. This may explain the ability for us to detect physiologically released ACh in vivo, and the challenge we encountered in brain slices. To detect optogenetically induced ACh release in brain slices, the brief duration of ACh transients demands a shorter integration time, resulting in fewer photons for lifetime estimates and a diminished signal-to-noise ratio (*81*). In contrast, in FLiP experiments in vivo, the collection of light from a larger number of cells leads to higher photon counts, resulting in an enhanced signal-to-noise ratio even at faster sampling rates. This study (Figs. 6 and 7) and others (*7*) demonstrate the capability of fluorescence lifetime to detect physiologically relevant signals with subsecond to second temporal resolution in vivo. Recent innovations in lifetime measurements have enabled higher sampling rate (*85*–*87*). Moreover, the lower sampling rate of lifetime measurements can be addressed by concurrent intensity measurement at a higher sampling rate. Notably, given the different EC_50_ values for intensity and lifetime measurements of the ACh sensor (Fig. 1G), simultaneous intensity and lifetime measurements offer the added advantage of expanding the sensitivity range of the sensor.

In summary, fluorescence lifetime excels over intensity when one needs to compare changes across individual animals, across fluctuating excitation light power, and across chronic time scale, and simultaneous intensity and lifetime measurements can expand sensitivity range of sensors and provide benefits of both methods.

### Opportunities for biological discoveries

Despite decades of research on neuromodulators, many questions remain. Notably, although recent findings reveal the importance of both tonic and phasic release of neuromodulators, it is unknown when tonic versus phasic change of neuromodulator release occurs during animal behavior. In addition, neuromodulators are released widely into many brain regions (*88*), but it is unclear whether their release is differentially regulated in different regions. Last, most drugs for psychiatric disorders target neuromodulators or their receptors (*13*, *16*, *17*, *89*–*92*), but we cannot easily compare neuromodulator levels between control and disease models and between pre-drug and post-drug periods, and we understand even less whether these drugs alter transient or sustained levels of neuromodulators. All these questions were hindered by the lack of a method to measure both transient and sustained change of neuromodulators simultaneously.

The discovery and demonstration of the power of fluorescence lifetime-based sensors open avenues for biological discoveries (Fig. 8). We demonstrate consistent in vivo lifetime measurement of neuromodulator concentrations across individual animals, imaging conditions, and chronic time scale (Figs. 6 and 7). Fluorescence lifetime can record neuromodulator dynamics across multiple time scales: On the fast end, it can resolve transient neuromodulator changes over subseconds; on the slow end, lifetime is stable over long periods of time and can therefore track slow biological processes happening across days, weeks, and months, when intensity loses its fidelity due to changing sensor expression level and variation of imaging conditions. Thus, our method enables dissection of transient and sustained neuromodulator changes between behavior states, between brain regions, and across aging. Furthermore, it allows us to disambiguate whether transient or sustained change of neuromodulator release is the predominant driver of disease conditions and in response to therapies. Thus, lifetime measurement of neuromodulators holds exciting potential for studying normal physiology, disease processes, and drug effects.

### Opportunities for sensor design

We report a method that can accurately measure both transient and sustained change of neuromodulators. Our discovery of lifetime response by GPCR-based single fluorophore sensors provides the foundation for developing more lifetime-based neuromodulator sensors. Current neuromodulator sensors have not been optimized for lifetime measurement because they have generally been selected for low intensity at baseline and not for lifetime response. Despite the lack of optimization for fluorescence lifetime measurement, lifetime of GRAB_ACh3.0_ shows high signal-to-noise ratio that is comparable to most FRET-based sensors and can be used to distinguish ACh between different behavior states in vivo (Figs. 6 and 7). In contrast, the sensors for DA, NE, and serotonin showed a lifetime change too small to be useful in practice (Fig. 1B). The connection between the magnitude of lifetime changes and the sequences of the sensors is indirect. On one hand, these differing responses highlight the surprise of lifetime change in GPCR-based single fluorophore sensors. On the other hand, they show future promise of turning intensity-based sensors into lifetime-based sensors by systematic mutagenesis and screening.

To optimize for lifetime response, sensors need to be screened for (i) increased brightness to make measurement of fluorescence lifetime reliable at all neuromodulator concentrations because autofluorescence can distort lifetime measurement when sensor brightness is low, (ii) lack of formation of aggregates because the difference in lifetime between aggregates and functional sensors (Fig. 3A) complicates the quantitation of absolute neuromodulator concentrations in photometry experiments in vivo, (iii) larger dynamic range between different neuromodulator concentrations, and (iv) minimal variation in lifetime readout with the same neuromodulator concentration between cells and between animals. Given the demonstrated power of fluorescence lifetime for comparison of transient and sustained neuromodulator changes across animals, between imaging conditions, and across chronic time periods, all sensor developers should consider fluorescence lifetime, in addition to intensity, as a criterion for sensor screening and optimization in the future.

## MATERIALS AND METHODS

### HEK 293T cells

HEK 293T cells were cultured in Dulbecco’s modified Eagle’s medium with 10% fetal bovine serum (Millipore Sigma), GlutaMAX (Invitrogen), and penicillin/streptavidin (50 U/ml; Corning) at 37°C in 5% CO_2_. All cells were female. The cell line has not been authenticated. They were plated on coverslips in 24-well plates and transfected with plasmids (0.4 to 0.8 μg per well) using Lipofectamine 2000 (Invitrogen). Two days after transfection, the cells were imaged with perfusion of artificial cerebrospinal fluid (ACSF; concentrations: 127 mM NaCl, 25 mM Na_2_CO_3_, 1.25 mM NaH_2_PO_4_·H_2_O, 2.5 mM KCl, 1 mM MgCl_2_, 2 mM CaCl_2_, and 25 mM glucose).

### Animals

All procedures for rodent husbandry and surgery were performed following protocols approved by the Washington University Institutional Animal Care and Use Committee and in accordance with National Institutes of Health guidelines. Either adult wild-type C57BL/6J mice (JAX, 000664) or *Emx1^IRES cre^* (JAX, 005628) mice were used.

### DNA plasmids

The constructs *pdisplay-CMV-GRAB_ACh3.0_* (*60*), *pdisplay-CMV-gGRAB_5-HT2h_* (*62*), *pdisplay-CMV-GRAB_NE2m_* (*63*), *pdisplay-GRAB_ACh3.0mut_* (*60*), and *pdisplay-GRAB_DA2m_* (*64*) were gifts from Y. Li’s laboratory. *pAAV-CAG-iAChSnFR* (Addgene, #137955) was from L. Looger’s laboratory (*61*).

### Virus production and stereotaxic injections

AAV9-hSyn-DIO-GRAB_ACh3.0_ (*60*) (DNA corresponding to Addgene, #121923) and AAV9-hSyn-GRAB_ACh3.0mut_ (*60*) viruses were packaged at Vigene Biosciences. AAV5-CamKII-Cre was from J. M. Wilson and packaged at Addgene (Addgene, #105558-AAV5). For stereotaxic injection, dorsal hippocampus CA1 was targeted with coordinates of posterior 1.78 mm and lateral 1.58 mm relative to Bregma and 1.36 mm from the pia. All injections were made at a rate of 100 nl/min through a UMP3 micro-syringe pump (World Precision Instruments) via glass pipette. For acute brain slice imaging, bilateral injections of 500 nl of AAV9-hSyn-DIO-GRAB_ACh3.0_ [3.1 × 10^12^ genome copies (GC)/ml] and AAV5-CamKII-Cre (3 × 10^12^ GC/ml) were made in wild-type mice. For FLiP experiments, 500 nl of AAV9-hSyn-DIO-GRAB_ACh3.0_ (3.9 × 10^12^ GC/ml) was injected into left hemispheres of *Emx1^IRES cre^* mice. For control experiments, 500 nl of AAV9-hSyn-GRAB_ACh3.0mut_ (3.1 × 10^12^ GC/ml) was injected into the left hemispheres of wild-type mice. Following virus injection, optical fibers, EEG/EMG implants, and headplates were placed.

### Implantation of optic fibers, EEG/EMG implants, and headplate

After stereotaxic injection and withdrawal of the glass pipette, an optical fiber (Doric Lenses, MFC_200/245-0.37_2.5mm_MF1.25_FLT) was inserted into the same injection site, at 0.05 mm above the viral injection site. The fiber was stabilized to the skull with glue. To implant the EEG and EMG implants, four stainless steel screws were inserted into the skull, with two above the cerebellum, one above the right hippocampus, and one above the right frontal cortex. The screws were wired to an EEG/EMG headmount (Pinnacle, 8402). Two EMG electrodes from the headmount were inserted into the neck muscle of the mice. A headplate was placed directly onto the skull. All the implants were secured to the skull with dental cement. An additional layer of dental cement with black paint was applied for lightproofing. All experiments were carried out at least 2 weeks after the surgery.

### Acute brain slice preparation

Mice were anesthetized with isoflurane followed by intracardial perfusion with cold *N*-methyl-d-glucamine (NMDG)–based cutting solution (concentrations: 92 mM NMDG, 2.5 mM KCl, 1.25 mM NaH_2_PO_4_, 30 mM NaHCO_3_, 20 mM Hepes, 25 mM glucose, 10 mM MgSO_4_, 0.5 mM CaCl_2_, 5 mM sodium ascorbate, 2 mM thiourea, and 3 mM sodium pyruvate) (*93*). Their brains were rapidly dissected out. Coronal sections (300 μm thick) were obtained with a vibratome (Leica Instruments, VT1200S) in cold NMDG-based cutting solution. After sectioning, slices were transferred to NMDG-based solution and incubated at 34°C for 12 min and then kept in Hepes-based holding solution (concentrations: 92 mM NaCl, 2.5 mM KCl, 1.25 mM NaH_2_PO_4_, 30 mM NaHCO_3_, 20 mM Hepes, 2 mM thiourea, 5 mM sodium ascorbate, 3 mM sodium pyruvate, 2 mM CaCl_2_, 2 mM MgSO_4_, and 25 mM glucose) at room temperature with 5% CO_2_ and 95% O_2_. Slices were then transferred to a microscope chamber, and ACSF was perfused at a flow rate of 2 to 4 ml/min for imaging.

### Histology of brain slices

After FLiP experiments, histology of each mouse brain was checked and only those with correct sensor expression and fiber implant location were used for further analyses. Mice were anesthetized with isoflurane, underwent intracardial perfusion with cold phosphate-buffered saline, followed by 4% paraformaldehyde (PFA). Their brains were harvested and placed in 4% PFA overnight at 4°C. Coronal slices (50 μm thick) were obtained with a vibratome (Leica Instruments, VT1200S). The slices were mounted with mounting media and then imaged with an epifluorescence microscope (Nikon E800). Images were taken by a camera (Teledyne Photometrics, CoolSnap EZ) and software QCapture Pro. Series of images were stitched using Fiji.

### 2pFLIM and image analysis

Two photon imaging was achieved by a custom-built microscope with a mode-locked laser source (Spectra-Physics, Insight X3 operating at 80 MHz). Photons were collected with fast photomultiplier tubes (PMTs, Hamamatsu, H10770PB-40). A 60× [Olympus, numerical aperture (NA) 1.1] or 20× (Nikon Fluor, NA 0.75) objectives were used for cellular resolution or whole field of view imaging, respectively. Image acquisition was performed with the custom-written software ScanImage (*94*) in MATLAB 2012b.

FLIM was performed as described previously (*45*, *46*). For all the green fluorescent protein–based neuromodulator sensors, 920 nm was used as the excitation wavelength. Emission light was collected through a dichroic mirror (FF580-FDi01-25X36, Semrock) and a band-pass filter (FF03-525/50-25, Semrock). The 128 × 128 pixel images were collected by frame scan at 4 Hz. The FLIM board SPC-150 (Becker and Hickl GmbH) was used, and time-domain single-photon counting was performed in 256 time channels. Photons from 20 frames were pooled for intensity and fluorescence lifetime calculation, which gave a sampling rate of ~0.2 Hz. For cellular resolution imaging, only healthy cells (judged by gradient contrast images) with membrane expression pattern were selected. Cells with round shape, sensor expression aggregates, or cell-filling expression patterns were excluded. The membrane of individual cells was selected as region of interest (ROI). To minimize the effect of movement artifact on intensity measurement, pixels with photon counts below 5 was omitted and then the top 66% brightest pixels were selected as effective pixels. Photons from effective pixels of a given ROI were pooled for further analysis. For whole field of view based FLIM analysis, pixels with more than 300 photons were excluded to avoid dead time artifact of the FLIM driver board. Photons from the rest of the pixels in the field of view were pooled for further analysis. The average photon count per pixel was used for intensity measurement. The average lifetime of all the photons in this ROI was calculated as follows

$$\tau =\frac{\sum \left[F\left(t\right)*t\right]}{\sum F\left(t\right)}$$

in which *F*(*t*) is the photon count from a certain fluorescence lifetime histogram time channel, and *t* is the lifetime measurement corresponding to the same time channel. We performed the calculation from 0.0489 to 11.5 ns in the lifetime histogram. Because of the change of cable length in FLIM or FLiP setup, the empirical lifetime across different experiments showed different absolute values. The cable length was kept consistent within one set of experiments.

Change of fluorescence lifetime at baseline was quantitated as lifetime measurement averaged over the first five data points of baseline subtracted from lifetime measurement averaged over the last five data points of baseline. Change of lifetime due to treatment was calculated as the average lifetime of the last five data points of baseline subtracted from that of the last five data points of treatment period. Cells with unstable baseline (coefficient of variation for baseline lifetime larger than 0.8%) were excluded. Similar calculations were performed for intensity change, with change of intensity divided by the average intensity of the first five data points of baseline as Δ*F*/*F*_0_.

For puffing experiments, imaging was performed at a sampling rate of ~0.7 Hz. Changes of fluorescence lifetime or intensity were quantitated as baseline measurement (average of the first 10 data points of baseline) subtracted from the maximum of a given period (baseline or puffing). Change of intensity was expressed as Δ*F*/*F*_0_. For dose-dependent response experiments, the response of each concentration of ACh treatment was expressed as the percentage of the peak responses.

### FLiP and analysis

A FLiP setup was custom built and used similar to that previously described (*83*). Briefly, a pulsed 473-nm laser (Becker and Hickl, BDS-473-SM-FBE operating at 50 MHz) was used as the excitation light source. An optical fiber patch cord (Doric Lenses, MFP_200/220/900–0.37_1.5m_FCM-MF1.25_LAF) was used to direct the excitation laser beam to the optical fiber implanted in the mouse brain. A dichroic mirror (Thorlabs, DMLP505R) and band-pass filter (Semrock, FF01-525/39-25) were used to select the green emission light from the blue excitation light. Emission light was detected with a fast PMT (Hamamatsu, H10770PA-40), and a time-correlated single-photon counting (TCSPC; SPC-150, Becker and Hickl GmbH) board was used to measure fluorescence lifetime binned into 256 time channels. The data were collected by customized software in MATLAB 2012b at 1 Hz. Excitation light power was adjusted with a neutral density filter, so the photon arrival rate was between 1 × 10^5^/s and 8 × 10^5^/s. The lower limit was chosen for accurate estimation of lifetime, and the upper limit chosen based on the dead time of the TCSPC driver board. The typical excitation power needed to generate the appropriate rate of photons for TCSPC was 0.01 to 0.18 μW (measured at the output end of the patch cord). Location of viral injection and fiber implants examined by histology after experiments. Only mice with tip of the fiber above hippocampus CA1 were used in the behavior analysis. For data analysis, we calculated average lifetime from 2.148 to 18.555 ns in the lifetime histogram.

### Running and resting recording and analysis

Mice with optic fiber implant and headplate were head-fixed on a treadmill and recorded in the dark. An incremental rotary encoder (SparkFun, COM-11102) was used to record the speed of the voluntary running. Rotary signals were collected at 25 Hz via an Arduino Due board (Arduino, A000062). The signals were sent to Bonsai (https://bonsai-rx.org/) via serial port communication and timestamped in Bonsai. Videos were simultaneously recorded at 25 frames per second (fps) in Bonsai. FLiP data were collected at 1 Hz.

Raw data of running speed were binned to 4 Hz for analysis. Running epochs were defined by the following criteria: (i) continuous forward or backward movement above a speed of 1 cm/s, (ii) no more than three consecutive subthreshold data points, (iii) preceded by at least 10 s of subthreshold resting, and (iv) at least 5 s in duration. For ACh sensor fluorescence analysis during running, to account for sensor kinetics, 3 s at the beginning of each running epoch was excluded for analysis. Each resting epoch was specified as continuous below-threshold speed that lasts for more than 150 s. To account for sensor kinetics and ACh kinetics, the first and last 30 s of each resting epoch were excluded for analysis. If a trimmed resting epoch is longer than 90 s, then it is split into 90-s epoch segments.

The median values of fluorescence intensity or fluorescence lifetime of ACh sensor for each running or resting segment were quantitated for subsequent analysis. For resting-to-running transition-related change, the median values of the fluorescence intensity or lifetime during −10 to −5 and − 5 to 0 s before the transition were quantitated as baseline start and baseline end, respectively. The differences between baseline end and baseline start were calculated as baseline changes. The differences between running and baseline end were calculated as resting→running changes.

### FLiP, EEG/EMG, and video recordings

Mice that underwent GRAB_ACh3.0_ virus injection, optical fiber implantation, and EEG/EMG implant were placed in a chamber with 12-hour/12-hour light-dark cycle (6 a.m. to 6 p.m. light). Recordings from 9 p.m. to 6 a.m. (dark phase) were collected and analyzed. An additional infrared light was used for video recording during the dark phase. Fluorescence lifetime and intensity data were collected at 1 Hz with our custom-built FLiP setup. EEG/EMG recording was performed at 400 Hz with a system from Pinnacle Technology using our ScanImage software. Video recording was performed at 25 fps in Bonsai. Video data were synchronized with FLiP and EEG/EMG data via a TTL (transistor-transistor logic) signal from MATLAB to Arduino Due board (Arduino, A000062) to Bonsai to trigger the start of video recording.

### Sleep stage scoring and analysis

Sleep stages were scored for every 4-s bin based on the EEG, EMG, and motion detection from the video using a custom-written program in Python. Briefly, sleep scoring prediction was generated with a random forest model, followed by user correction. The following criteria were used to determine sleep/wake stages (*60*, *95*): (i) AW: low variance in EEG, high variance in EMG, and high movement based on video; (ii) quiet wakefulness: low variance in EEG, low variance in EMG, and low movement based on video; (iii) NREM sleep: high variance in EEG with high delta power (0.5 to 4 Hz), low variance in EMG, and no movement based on video; (iv) REM sleep: high theta (5 to 8 Hz) to delta power ratio based on EEG, low variance in EMG, and no movement based on video.

For quantification of ACh sensor measurement in a given behavior epoch, to minimize the effect of kinetics of the sensor or behavior state-related ACh change, epochs longer than 40 s were included, and within each epoch, 12 s were trimmed at each end with the middle portion used for subsequent analyses. The median values of ACh sensor measurement in each epoch were quantitated for subsequent analysis. To quantify ACh change upon NREM to REM sleep transitions, transition events with at least 50 s of NREM sleep before transition time were included. The median values of ACh measurements from −50 to −35 s were quantified as baseline start. The baseline end and transition response were defined as the median values of ACh sensor measurements during the equilibrium period before (from −35 to −20 s) and after (from 20 to 35 s) NREM-REM transition time. The differences between baseline end and baseline start and between transition response and baseline end were quantified as baseline change and NREM→REM transition-related change. For quantitation of intensity change Δ*F*/*F*_0,_ *F*_0_ was the average photon count across the whole recording.

### Pharmacology

Unless otherwise noted, all chemicals were applied via bath perfusion: They were either added to the perfusion reservoir or premade buffers with the specified chemicals were switched from one to another. Lifetime was allowed to stabilize before a chemical was added. When there was no clear lifetime change, 10 min was recorded before the addition of another chemical or the end of the experiment. The final concentrations of chemicals are specified in parentheses: ACh chloride (0.001 to 100 μM), NE bitartrate monohydrate (10 μM), and DA hydrochloride (10 μM) were from Sigma-Aldrich; serotonin hydrochloride (5-HT; 100 μM), mAChR antagonist tiotropium bromide (Tio; 5 μM), and cholinesterase inhibitor donepezil hydrochloride (5 μM) were from Tocris. For puffing experiments, a glass patch pipette was used to locally puff ACh (200 μM in ACSF) for 10 s onto a neuron in a brain slice through a Picospritzer (Parker, 052-0500-900) at 2 psi.

### FLIM simulation

The simulation was performed by customized MATLAB code, and the simulation procedures and codes were described in detail in (*81*). For the simulation in this study, the null hypothesis is that with or without ACh binding, GRAB_ACh3.0_ has the same fluorescence lifetime and can be described by the same equation—thus, the apparent fluorescence lifetime change was solely due to altered proportion of autofluorescence contribution. The simulated lifetime distribution includes photons from multiple sources. (i) The fluorescence of GRAB_ACh3.0_ was modeled by a double exponential decay.

$$F=F_{0}\cdot \left[p_{1}\cdot e^{\left(-\frac{t}{\tau_{1}}\right)}+p_{2}\cdot e^{\left(-\frac{t}{\tau_{2}}\right)}\right]$$

τ1, τ2, *p*1, and *p*2 were determined empirically by measuring the fluorescence decay of ACh 3.0 expressed in HEK cells at saturating concentration (100 μM) of ACh. A large population of photons (~6 × 10^6^) with specific lifetimes was generated on the basis of the double exponential decay and binned into 256 time channels over 12.5 ns (time interval between laser pulses for an 80-MHz laser). To simulate lifetime measurements across cells, a small sample of photons was drawn with replacement from the large population, and the number of photons in the sample corresponded to the average of measured photons at either 0 or 100 μM of ACh, respectively. To simulate noise from the instruments, the lifetime of a specific photon from the sample was then transformed into a convolved lifetime based on random draw from the distribution of a pulse response function (PRF). The PRF was measured empirically with second harmonic generation of collagen fibers with mouse tails. (ii) We added photons due to afterpulse (0.32% of total photon count that is measured empirically, with even distribution across lifetime). (iii) Lifetime of photons due to autofluorescence were sampled with replacement from empirically determined autofluorescence distribution, produced through imaging of untransfected HEK 293T cells. Simulation was repeated 500 times for each sample size corresponding to 0 or 100 μM ACh. Empirical fluorescence lifetime was calculated for each simulated combination and compared to experimentally observed values.

### Quantification and statistical analysis

Detailed information of the quantification, sample size, and statistics used are summarized in figure legends, figures, and Results. Wilcoxon test (with Bonferroni correction when appropriate) was performed for paired data. Mann-Whitney test was performed for unpaired data. Dose-response curves were fitted to an asymmetrical generalized Hill equation model to calculate the EC_50_. For analysis of variance, Friedman test was performed for matched data, and Kruskal-Wallis test was performed for unmatched data, followed by Dunn’s multiple comparison [one-way analysis of variance (ANOVA)], or Šídák’s multiple comparison (two-way ANOVA). Nested *t* test or one-way ANOVA was performed when comparison was made with hierarchical data. Two-way ANOVA was used to determine the contribution to the total variance from two independent variables. All these statistical analyses were performed in GraphPad Prism 9.

GLM was used to analyze the correlation between independent variable and dependent variable in MATLAB. For S6E and S6F, GLM was applied with the independent variables being running speed or duration, mouse ID, and laser power. For Figs. 6G and 7H, a stepwise-GLM model was performed in MATLAB to determine the contribution to the total variance. The independent variables were added in order of weights (largest first based on adjusted *R*^2^), and the subsequent improvement to overall adjusted *R*^2^ was calculated as the contribution to the variance for each independent variable.

Logistic regression (LR) was used to identify the strength of the relationship of individual independent variables (intensity and lifetime) on states (resting/running; REM/NREM). LR was performed using Scikit-Learn in Python. McFadden’s pseudo-*R*^2^ values were used to evaluate the performance of the model.
