# Supplementary material for: Fast and slow: Recording neuromodulator dynamics across both transient and chronic time scales
Source: Sci Adv. 2024 Feb 21;10(8):eadi0643. doi: 10.1126/sciadv.adi0643 (PMC10881037; doi:10.1126/sciadv.adi0643)
Supplement: Supplementary file 1 — Figs. S1 to S7 [file sciadv.adi0643_sm.pdf]

Science Advances
AAAS

Supplementary Materials for

Fast and slow: Recording neuromodulator dynamics across both transient and chronic time scales

Pingchuan Ma *et al.*

Corresponding author: Yao Chen, yaochen@wustl.edu

Sci. Adv. **10**, eadi0643 (2024)
DOI: 10.1126/sciadv.adi0643

This PDF file includes:

Figs. S1 to S7

Figure S1

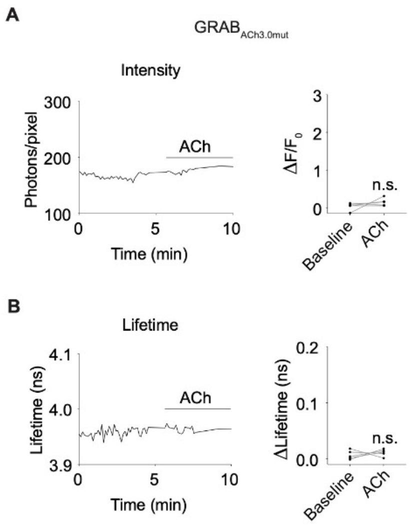
A
GRAB$_{ACh3.0mut}$
Intensity
Photons/pixel
300
200
100
ACh
0
5
10
Time (min)
3
2
1
0
ΔF/F$_0$
n.s.
Baseline
ACh
B
Lifetime
Lifetime (ns)
4.1
4.0
3.9
ACh
0
5
10
Time (min)
0.2
0.1
0.0
ΔLifetime (ns)
n.s.
Baseline
ACh

Figure S1. GRAB$_{ACh3.0mut}$ sensor showed no fluorescence lifetime or intensity change to ACh application.
(A-B) Traces (left) and summaries (right) of intensity (A) and fluorescence lifetime (B) response of GRAB$_{ACh3.0mut}$ sensor to ACh application (100 μM). Wilcoxon test, n.s., not significant vs baseline.

Figure S2

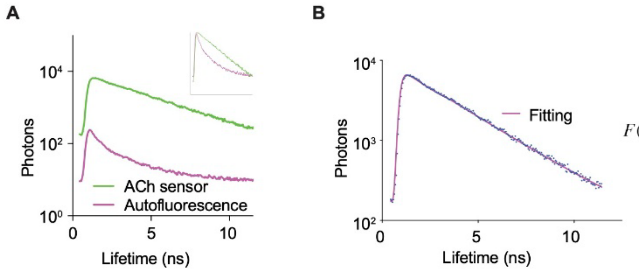

$$F(t) = F_0\left[p_1 \cdot e^{\left(-\frac{t}{\tau_1}\right)} + p_2 \cdot e^{\left(-\frac{t}{\tau_2}\right)}\right]$$

τ_1=2.8808 ns, τ_2=0.4631ns
p_1=0.9046, p_2=0.0954

Figure S2. Fluorescence lifetime of sensor fluorescence and autofluorescence.
(A) Measured histogram of fluorescence lifetime of HEK293T cells without sensor expression (autofluorescence), and with $GRAB_{ACh3.0}$ expression (in the presence of 100 μM ACh). The inset shows histogram normalized to peak photon count, indicating that the fluorescence lifetime of autofluorescence is shorter than sensor fluorescence.
(B) Measured histogram and corresponding double exponential curve fitting results of fluorescence lifetime of $GRAB_{ACh3.0}$ in HEK293T cells in the presence of 100 μM ACh.

Figure S3

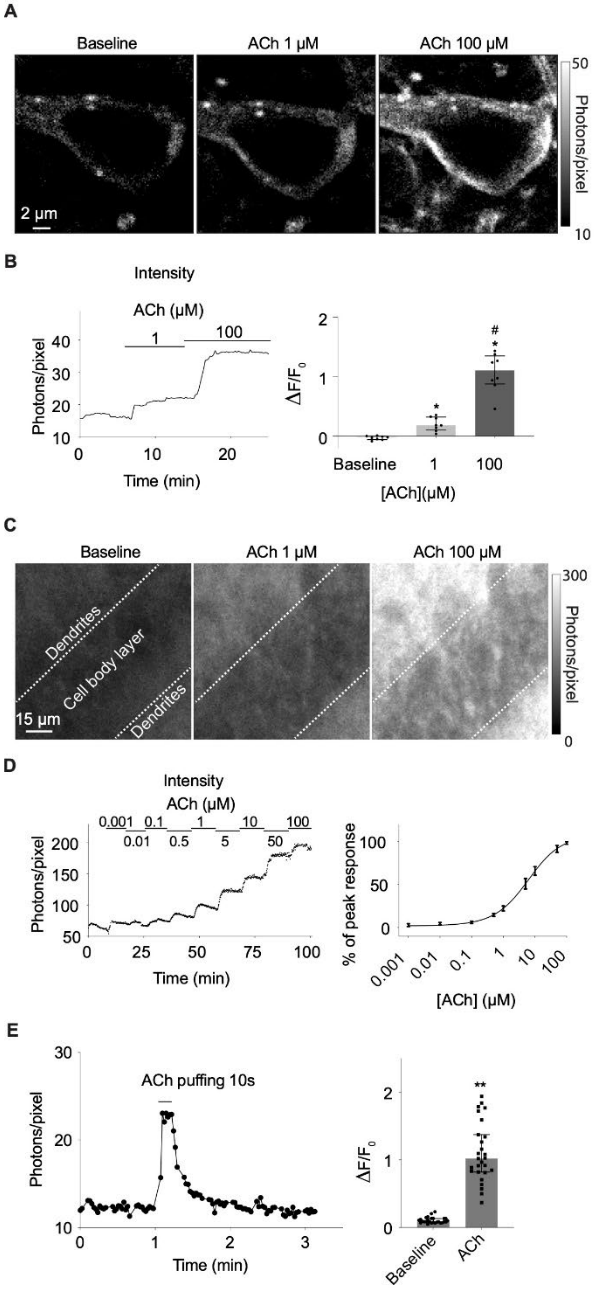
A
Baseline
ACh 1 μM
ACh 100 μM
50
Photons/pixel
10
2 μm
B
Intensity
ACh (μM)
1
100
Photons/pixel
Time (min)
ΔF/F0
Baseline
1
100
[ACh](μM)
C
Baseline
ACh 1 μM
ACh 100 μM
Dendrites
Cell body layer
Dendrites
15 μm
300
Photons/pixel
0
D
Intensity
ACh (μM)
0.001
0.01
0.1
0.5
1
5
10
50
100
Photons/pixel
Time (min)
% of peak response
[ACh] (μM)
E
ACh puffing 10s
Photons/pixel
Time (min)
ΔF/F0
Baseline
ACh

Figure S3. Intensity of GRAB$_{ACh3.0}$ responds to graded transient ACh in brain tissue.
(A-B) Heatmaps (A), example trace, and summaries (B) showing fluorescence intensity changes of individual hippocampal CA1 pyramidal neurons expressing GRAB$_{ACh3.0}$ in response to ACh (1 μM and 100 μM, with 5 μM AChEi Donepezil). Wilcoxon test with Bonferroni correction, *adjusted $p < 0.05$ vs baseline, $^{\#}$adjusted $p < 0.05$ vs 1 μM. Data are represented as median with interquartile range.
(C-D) Heatmaps (C), example trace and dose-response curve (D) of fluorescence intensity of 90 μm x 90 μm fields of views of hippocampal CA1 neurons expressing GRAB$_{ACh3.0}$ in response to various concentrations of ACh (with 5 μM AChEi Donepezil). Data in the dose-response curve are represented as mean with SEM.
(E) Example trace and summaries showing fluorescence intensity of GRAB$_{ACh3.0}$ in CA1 pyramidal neurons in response to a 10-second puff of ACh (200 μM). Wilcoxon test, $^{**}p < 0.01$ vs baseline. Data are represented as median with interquartile range.

Figure S4

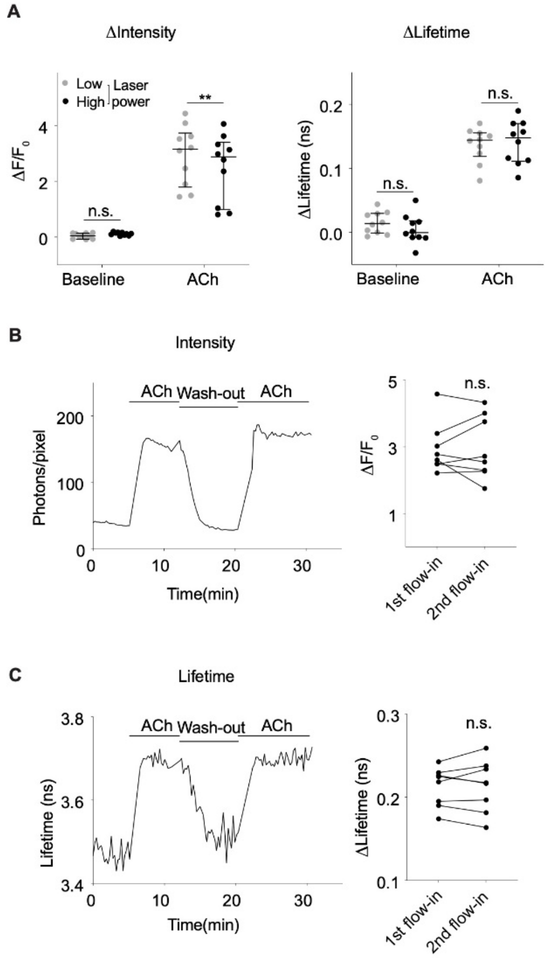
A
ΔIntensity
ΔLifetime
Low
High
Laser power
ΔF/F0
**
n.s.
Baseline
ACh
ΔLifetime (ns)
B
Intensity
ACh
Wash-out
ACh
Photons/pixel
Time(min)
ΔF/F0
1st flow-in
2nd flow-in
C
Lifetime
Lifetime (ns)
ΔLifetime (ns)

Figure S4. Fluorescence lifetime, and relative change of fluorescence lifetime of $GRAB_{ACh3.0}$ is stable with repeated ACh application and across laser powers.

(A) Summaries of intensity and fluorescence lifetime changes of individual 293T cells expressing $GRAB_{ACh3.0}$ in response to ACh (100 μM, with 5 μM of AChEi Donepezil) under different laser powers. Two-way ANOVA with Šídák's multiple comparison, $^{**}p < 0.01$, n.s., not significant, low vs high laser power.

(B-C) Trace and summaries of intensity (B) and fluorescence lifetime (C) measurement of $GRAB_{ACh3.0}$ in HEK 293T cells in response to repeated flow-in of the same concentration of ACh (1 μM, with 5 μM of AChEi Donepezil). Wilcoxon test; n.s., not significant vs 1st flow-in.

Data are represented as median with interquartile range.

Figure S5

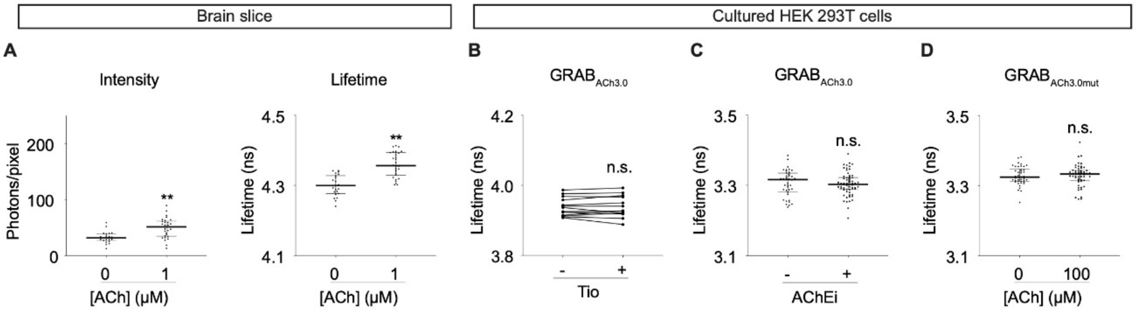

Brain slice
Cultured HEK 293T cells
A
Intensity
Lifetime
B
GRAB ACh3.0
C
GRAB ACh3.0
D
GRAB ACh3.0mut
Photons/pixel
200
100
0
**
0
1
[ACh] (μM)
4.5
4.3
4.1
Lifetime (ns)
**
0
1
[ACh] (μM)
4.2
4.0
3.8
Lifetime (ns)
n.s.
-
+
Tio
3.5
3.3
3.1
Lifetime (ns)
n.s.
-
+
AChEi
3.5
3.3
3.1
Lifetime (ns)
n.s.
0
100
[ACh] (μM)

Figure S5. Distribution of fluorescence lifetime of $GRAB_{ACh3.0}$ and $GRAB_{ACh3.0mut}$.
(A) Distribution of fluorescence intensity and lifetime of hippocampal CA1 pyramidal neurons from acute brain slices expressing $GRAB_{ACh3.0}$ with or without ACh (1 μM, with 5 μM of AChEi Donepezil). Wilcoxon test, $^{**}p < 0.01$, vs baseline.
(B) Fluorescence lifetime of $GRAB_{ACh3.0}$ in HEK 293T cells before and after application of mAChR antagonist Tiotropium (5 μM). Wilcoxon test, n.s., not significant, vs baseline.
(C) Distribution of fluorescence lifetime of $GRAB_{ACh3.0}$ in HEK 293T cells with or without AChEi Donepezil (5 μM). Mann-Whitney test, n.s., not significant vs without AChEi.
(D) Distribution of fluorescence lifetime of $GRAB_{ACh3.0mut}$ sensor in HEK 293T cells with or without ACh (100 μM). Mann-Whitney test, n.s., not significant, vs without ACh.
Data are represented as median with interquartile range.

Figure S6

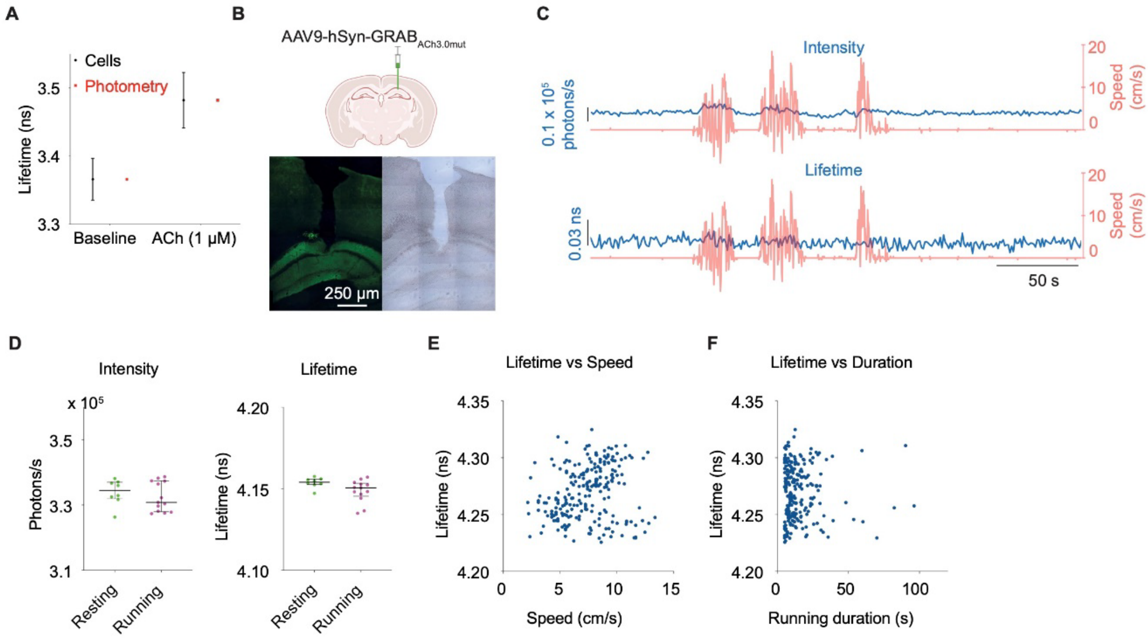

A
Cells
Photometry
Lifetime (ns)
3.5
3.4
3.3
Baseline
ACh (1 μM)
B
AAV9-hSyn-GRAB ACh3.0mut
250 μm
C
Intensity
0.1 x 10^5 photons/s
Speed (cm/s)
20
10
0
Lifetime
0.03 ns
50 s
D
Intensity
x 10^5
Photons/s
3.5
3.3
3.1
Resting
Running
Lifetime
Lifetime (ns)
4.20
4.15
4.10
E
Lifetime vs Speed
4.35
4.30
4.25
4.20
0
5
10
15
Speed (cm/s)
F
Lifetime vs Duration
0
50
100
Running duration (s)

Figure S6. Intensity and fluorescence lifetime measurements of GRAB$_{ACh3.0mut}$ sensor in the hippocampus during running/resting.

(A) Schematic illustrating the reduction in standard deviation of data in bulk measurement with FLiP compared with cell-based imaging. Photometry data were modelled based on light collection from 1000 cells. Data are represented as mean with standard deviation.

(B) Illustration and histology images of the green (left) and phase contrast (right) channels showing expression of GRAB$_{ACh3.0mut}$ in CA1 cells of hippocampus, and implantation of optical fibers into the CA1 region of the hippocampus. AAV carrying *GRAB$_{ACh3.0mut}$* driven by neuronal specific hSyn promoter was delivered to CA1 cells in the hippocampus of wild-type mice. The schematic illustration was created with BioRender.

(C) Example traces showing intensity (top, blue) or fluorescence lifetime (bottom, blue) measurements from FLiP, and running speed (red) of GRAB$_{ACh3.0mut}$-expressing mice on a treadmill.

(D) Distribution of intensity and fluorescence lifetime of GRAB$_{ACh3.0mut}$ sensor in resting or running states of one mouse. Data are represented as median with interquartile range.

(E) Correlation between the median lifetime of GRAB$_{ACh3.0}$ and running speed of each running epoch.

(F) Correlation between the median lifetime of GRAB$_{ACh3.0}$ and running duration of each running epoch.

Figure S7

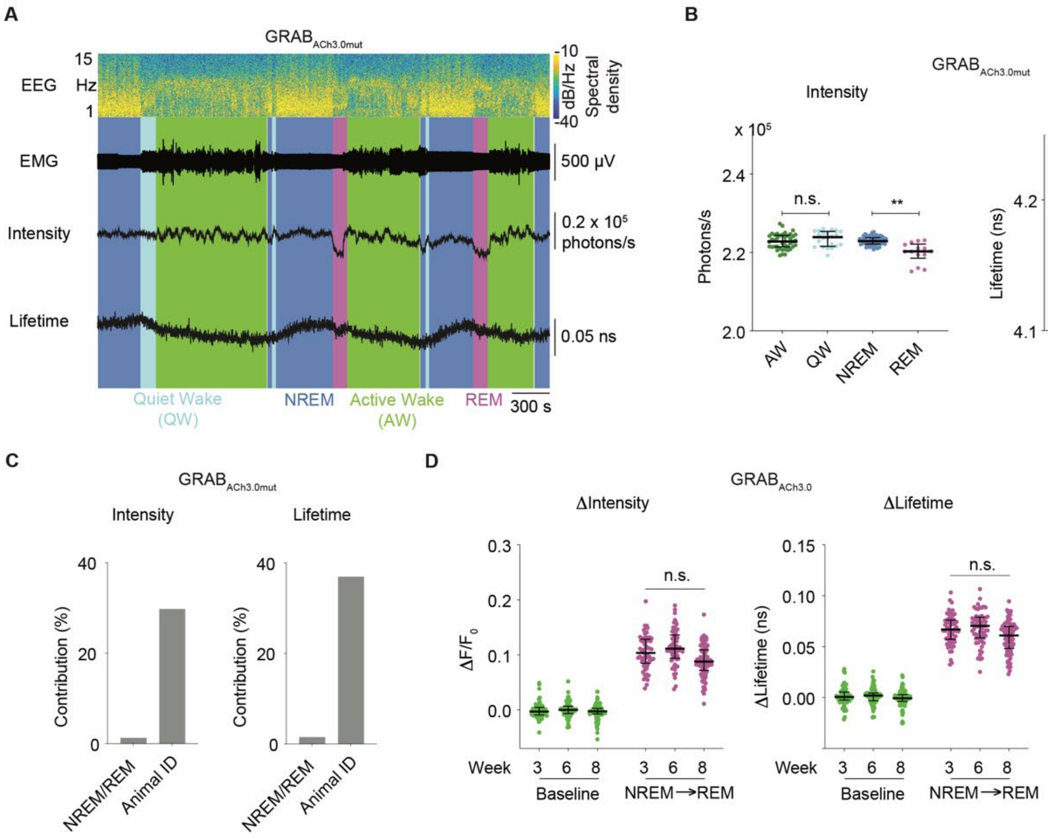
A
GRAB ACh3.0mut
EEG
15
Hz
1
-10
dB/Hz
-40
Spectral density
EMG
500 µV
Intensity
0.2 x 10⁵ photons/s
Lifetime
0.05 ns
Quiet Wake (QW)
NREM
Active Wake (AW)
REM
300 s
B
GRAB ACh3.0mut
Intensity
Lifetime
x 10⁵
Photons/s
Lifetime (ns)
n.s.
**
AW
QW
NREM
REM
C
GRAB ACh3.0mut
Intensity
Lifetime
Contribution (%)
NREM/REM
Animal ID
D
GRAB ACh3.0
ΔIntensity
ΔLifetime
ΔF/F0
ΔLifetime (ns)
Week
Baseline
NREM→REM

Figure S7. Stability of intensity and fluorescence lifetime changes over time, and measurements of $GRAB_{ACh3.0mut}$ sensor in the hippocampus across sleep-wake cycles.
(A) Example of spectrogram of EEG recording, EMG trace, the corresponding scored sleep-wake states, along with intensity and fluorescence lifetime traces of $GRAB_{ACh3.0mut}$ sensor from a mouse within 1 hour. Note the decrease in intensity during REM state.
(B) Summary of intensity and fluorescence lifetime measurements of $GRAB_{ACh3.0mut}$ sensor in different sleep-wake states. Kruskal-Wallis test with Dunn's multiple comparison, **adjusted $p < 0.01$; n.s., not significant. Data are represented as median with interquartile range.
(C) Results from two-way ANOVA analysis showing the contribution to the total variance of the measurements from the mutant ACh sensor due to behavior states (NREM vs REM) or animal identities. Note that behavior state (NREM or REM) gives minimal contribution to the total variance of the measurements.
(D) Summaries of the distribution of intensity and fluorescence lifetime changes of $GRAB_{ACh3.0}$ during NREM-REM transitions from recordings of 6 mice at different sensor expression time. Nested one-way ANOVA; n.s., not significant across sensor expression time. Data are represented as median with interquartile range.
